# A proximity proteomics screen in three-dimensional spheroid cultures identifies novel regulators of lumen formation

**DOI:** 10.1038/s41598-021-02178-2

**Published:** 2021-11-23

**Authors:** Li-Ting Wang, Marie-Ève Proulx, Anne D. Kim, Virginie Lelarge, Luke McCaffrey

**Affiliations:** 1 Rosalind and Morris Goodman Cancer Institute, McGill University, Montreal, QC H3A 1A3 Canada; 2 Division of Experimental Medicine, McGill University, Montreal, QC H4A 3J1 Canada; 3 Department of Biochemistry, McGill University, Montreal, QC H3G 1Y6 Canada; 4 Gerald Bronfman Department of Oncology, McGill University, Montreal, QC H4A 3T2 Canada

**Keywords:** Apicobasal polarity, Cellular imaging

## Abstract

Apical-basal cell polarity and lumen formation are essential features of many epithelial tissues, which are disrupted in diseases like cancer. Here, we describe a proteomics-based screen to identify proteins involved in lumen formation in three-dimensional spheroid cultures. We established a suspension-based culture method suitable for generating polarized cysts in sufficient quantities for proteomic analysis. Using this approach, we identified several known and unknown proteins proximally associated with PAR6B, an apical protein involved in lumen formation. Functional analyses of candidates identified PARD3B (a homolog of PARD3), RALB, and HRNR as regulators of lumen formation. We also identified PTPN14 as a component of the Par-complex that is required for fidelity of apical-basal polarity. Cells transformed with KRAS^G12V^ exhibit lumen collapse/filling concomitant with disruption of the Par-complex and down-regulation of PTPN14. Enforced expression of PTPN14 maintained the lumen and restricted the transformed phenotype in KRAS^G12V^-expressing cells. This represents an applicable approach to explore protein–protein interactions in three-dimensional culture and to identify proteins important for lumen maintenance in normal and oncogene-expressing cells.

## Introduction

Cell polarity is a fundamental property of epithelial cells that is crucial for their organization and homeostasis. The establishment of apical-basal polarity allows epithelial cells to adopt different structures in which the apical membranes are juxtaposed in neighbouring cells. For example, epithelial cells cultured on flat 2D surfaces form polarized monolayers with the apical membrane exposed to culture medium, whereas they organize into single layered acini with a central lumen when cultured in three-dimensional (3D) extracellular matrix^1^. Three dimensional cultures have multiple characteristics that differ from 2D cultures, including greater viability, more resistance to drug treatments, lower stiffness, and distinct metabolic profiles^1,2^. The differences in cell organization in 2D and 3D cultures are accompanied by changes in gene expression, with some genes involved in lumen formation upregulated in 3D environments^3–5^. 3D culture systems, therefore, offer many advantages and offer great potential for understanding development of tissue organization in normal and disease contexts.

Efficient induction of apical-basal polarity and formation of a central lumen require the coordinated activity of transmembrane proteins, scaffolds/adaptors, kinases, and GTPases that control vesicular trafficking^6,7^. Apical identity is directed by two polarity complexes: (1) the Par complex (PAR3, PAR6, and aPKC) at tight junctions regulates many aspects of epithelial organization, including apical-basal polarity, tight junction formation, cell division orientation, and cell migration^6^; and (2) the Crumbs complex (CRB3, PALS1, PATJ) which can also include PAR6 and aPKC^8,9^. PARD3 directly binds PAR6 and aPKC and recruits them to tight junctions through an association with JAMA to regulate tight junction formation and cell division orientation in 3D cysts, with diverse roles in vivo during development and cancer progression^10^. PARD3B is a homologue of PARD3 that has a similar domain structure; however, studies have indicated that unlike PARD3, PARD3B does not interact with aPKC and the association with PAR6B is controversial^11,12^. The function of PARD3B in epithelial cell polarity is unclear. PARD3B is highly expressed in the kidney, lung, and skeletal muscle, and is localized at tight junctions with tight junction protein ZO-1^12^. Ectopic expression of the N-terminal region of PARD3B disrupts the formation of tight junctions in MDCK cells^11^. Recent studies indicate that PARD3B binds to tumor suppressor protein Lkb1 and suppresses its kinase activity, whereas ablation of PARD3B causes rapid and profound stem cell loss that is vital for mammary gland stem cell maintenance^13^.

PAR6 is a dynamic polarity regulator that associates with multiple polarity proteins including aPKC, LLGL, PALS1, CRB3, and PARD3, and is required for efficient lumen formation in epithelial cells in 3D culture^14^.

Cell polarity proteins play a fundamental role in regulating many aspects of epithelial growth control and maintaining apical-basal polarity by controlling the localization of key mediators involved in regulating stem cell renewal, proliferation, apoptosis, survival, differentiation, cell motility, cell adhesion, and tissue organization, which are processes involved in both development and cancer progression^6^. Altered epithelial cell polarity is associated with development of carcinoma, characterized by collapse or filling of the lumen that accompanies epithelial overgrowth^15^. KRAS is one of the most frequently mutated genes (21%) across all cancers, with the highest prevalence in pancreatic, colorectal, endometrial, biliary tract, lung, and cervical cancers^16–18^. Recurrent point mutations at codon 12 of KRAS are frequent and produce a constitutively activate protein^16,19^. Several studies have demonstrated a relationship between KRAS and polarity. For example, mutations in the *KRAS* disrupt apical-basal polarity through inhibiting normal glycosylation of the β1-integrin chain of the collagen receptor in colon epithelial cells^20^. Activated KRAS also involves loss of apical-basal polarity and luminal cavity formation with apoptosis^21^. Previous studies indicate that PRKCI is required for Ras-induced transformation and tumorigenesis^22,23^.

Proximity-dependent biotin identification (BioID) was developed to characterize protein–protein interaction networks to screen a wide range of proteins and investigate protein networks and functions^24–28^. BioID is based on proximity-dependent cellular biotinylation by fusing a biotin ligase to a protein-of-interest in living cells. The biotinylated proteins are isolated using streptavidin affinity purification and can be analyzed by mass spectrometry^29^. Whereas wild-type BirA specifically biotinylates acetyl-CoA carboxylase through releasing a primed bioAMP molecule, which covalently attaches to a specific lysine of its substrate peptide^30,31^, modification of the biotin ligase BirA from *Escherichia coli* (R118G, BirA*) allows promiscuous biotinylation of proximal proteins (~ 10 nm radius), irrespective of whether they directly or indirectly interact, by generating highly reactive and short-lived bioAMP^29^. Since the proximal proteins are attached by stable covalent modification, harsh lysis conditions can be applied to solubilize most proteins^32^. Moreover, promiscuous biotin ligase (BioID2) from *A. aeolicus* is a smaller, more biotin-sensitive biotin ligase, thus improving on the original BioID^33^. This technique will allow for understanding of signaling networks in previously inaccessible biological settings including 3D organotypic skin cultures and in vivo^34,35^. However, it has been challenging to apply BioID to 3D spheroid cultures that are embedded or semi-embedded in basement membrane extracts due to the low-throughput nature of these culture conditions.

Here, we present optimized conditions to support efficient lumen formation of 3D spheroids in high-yield suspension cultures. We then applied BioID2 to identify PAR6B-proximity proteins as potential regulators of apical-basal polarity and lumen formation. We functionally validated PARD3B, RALB, and HRNR as regulators of lumen formation in Caco2-cysts. Moreover, we revealed a role for PTPN14 as a tumour suppressor that regulates cell polarity and epithelial organization in KRAS^G12V^-transformed epithelial cells. Our results demonstrate that 3D spheroid cultures are amenable to proteomics studies and that proximity-based methods can identify novel regulators of lumen formation.

## Methods

### Cell culture

Cell culture was performed as previously described^36^. Human intestinal epithelial cell line Caco-2 cells were purchased from American Type Culture Collection (ATCC). Caco-2 cells were cultured at 37 °C in 5% CO_2_ in DMEM (Wisent #319-005-CL) supplemented with 10% fetal bovine serum (Wisent #080-150), 100 U/ml penicillin, 0.1 mg/ml streptomycin (Wisent #450201EL). Human embryonic kidney cell line HEK293LT (ATCC) were cultured at 37 °C in 5% CO_2_ in DMEM supplemented with 10% fetal bovine serum, 100 U/ml penicillin, and 0.1 mg/ml streptomycin. For semi-embedded 3D cell culture, Caco-2 cells were seeded in 8 well μ-slide (Ibidi #80826) on top of a layer of 100% GelTrex (ThermoFisher Scientific #A1413202) in media supplemented 2% GelTrex at 37 °C under humidified atmosphere of 5% CO_2_. GelTrex is a growth factor basement membrane matrix extract (BME) that contains laminin, collagen IV, entactin, and heparin sulfate proteoglycans, which mimic the basement membrane context. For suspension-cell culture, cells were seeded on polyHEMA-coated plate in media supplemented with 2% GelTrex at 37 °C under humidified atmosphere of 5% CO_2_. For some experiments, the concentration of GelTrex was varied from 0 to 2%^36^.

To obtain sufficient cells and spheroids for mass spectrometry analysis, one million cells were seeded on four 15 cm plates in 2D or 3D cell culture. 2D or 3D cells were collected after 48 h of incubation with 50 μM biotin. Biotinylated proteins were lysed and isolated by binding to streptavidin beads and identified by mass spectrometry^36^.

### DNA constructs

mCherry, PAR6B, PRKCI, and EFGP were amplified by PCR from cDNA. pcDNA3.1 mycBioID was a gift from Kyle Roux (Addgene plasmid # 35700). myc-BioID2-MCS was a gift from Kyle Roux (Addgene plasmid # 74223). pWPI was a gift from Didier Trono (Addgene plasmid #12254). The PCR products were digested and inserted into myc-BioID2-MCS or pcDNA3.1 mycBioID. Fused products were digested and inserted into pWPI for virus production. Plasmids were verified by DNA sequencing. The pLX317-PTPN14-V5 plasmids were purchased from Sigma (TRCN, PTPN14 TRCN0000479328).

shRNAs targeting human *PARD3B*, *RALB*, *HRNR, and PTPN14* mRNA were cloned into pLKO. The shRNA used were acquired from the McGill Platform for Cellular Perturbation (MPCP).

sh3PARD3B CCGGCCATGCTTTGAGAACTGTCAACTCGAGTTGACAGTTCTCAAAGCATGGTTTTTG sh4PARD3B CCGGCCTGGTTACTGGGTGAAGATTCTCGAGAATCTTCACCCAGTAACCAGGTTTTTG, sh1RALB CCGGCGTGATGAGTTAAAGTTGTATCTCGAGATACAACTTTAACTCATCACGTTTTTG, sh5RALB CCGGGAGTTTGTAGAAGACTATGAACTCGAGTTCATAGTCTTCTACAAACTCTTTTTG, sh2HRNR CCGGGCTTTAGTCAACACAAGTCTACTCGAGTAGACTTGTGTTGACTAAAGCTTTTTTG, sh5HRNR CCGGGCAGCGGTAGTGTCTTTACTTCTCGAGAAGTAAAGACACTACCGCTGCTTTTTTG, sh1PTPN14 CCGGAGAGTCACCTCCAGACAACATCTCGAGATGTTGTCTGGAGGTGACTCTTTTTT, sh2PTPN14 CCGGGCGGTAATATACAGGTGGAATCTCGAGATTCCACCTGTATATTACCGCTTTTT, sh3PTPN14 CCGGCGGGAAGAGAATCGAGTTGATCTCGAGATCAACTCGATTCTCTTCCCGTTTTT, sh4PTPN14 CCGGCGCTCAGTACAAGTTTGTCTACTCGAGTAGACAAACTTGTACTGAGCGTTTTT, sh5PTPN14 CCGGCCTCAGAGAGTATGTGCTATTCTCGAGAATAGCACATACTCTCTGAGGTTTTT and a non-targeting scrambled shRNA was used as a control.

### Transient transfection

Transient transfections were performed as previously described^36^. HEK293LT cells were seeded at 2 × 10^6^ cells per well in 100 mm dishes and transfected with plasmids using Polyethylenimine (PEI) as per manufacturer's instructions (Sigma # 408727). Caco-2 cells were seeded at 4 × 10^4^ cells per well in 24 well and transfected with plasmids using Lipofectamine LTX as per manufacturer's instructions (Invitrogen #15338030). All experiments were performed 24 h post‐transfection^36^.

### Lentivirus production

Lentivirus was produced as previously described^36^. Lentivirus was produced by calcium phosphate transfection of HEK293LT cells in 15-cm dishes using 50 μg of lentiviral plasmid, 37.5 μg of packaging plasmid (psPAX2), and 15 μg of VSVG coat protein plasmid (pMD2.G). Viral supernatants were collected after 48 h. Viral supernatants were concentrated by precipitation in 40% polyethylene glycol 8000 (Bioshop # PEG800.1), followed by centrifugation, and then re-suspended in the culture medium. Concentrated virus was aliquoted and frozen at − 80 °C then titred using HEK293LT cells^36^.

### Affinity capture of biotinylated proteins

Biotinylated proteins were affinity captured as previously described^36^. Biotinylation was induced by adding 50 μM biotin for 48 h in cell cultures. After two PBS washes, cells were lysed in 600 μl ice cold RIPA lysis buffer (50 mM Tris–HCl (pH 7.5), 150 mM NaCl, 1% NP-40, 1 mM EDTA, 1 mM EGTA, 0.1% SDS, 0.5% sodium deoxcycholate, PMSF (1 mM), DTT (1 mM) and Sigma protease inhibitor cocktail (P8340, 1:500)). The lysates were treated with Benzonase for 1 h on ice, and an equal volume of RIPA lysis buffer was added. For each sample, 30 μl of streptavidin-sepharose bead slurry (GE Healthcare, Cat 17-5113-01) was pre-washed three times with 1 mL of lysis buffer by pelleting the beads with 400 g centrifugation and aspirating off the supernatant before adding the next wash. After three sessions of sonication and centrifugation at 16,500 g, supernatants with biotinylated proteins were incubated with pre-washed streptavidin beads for 3 h at 4 °C with rotation. Beads were collected and washed twice with RIPA buffer and thrice with 1 mL with 50 mM ammonium bicarbonate (pH 8.0). Beads were then resuspended in 100 mL of 50 mM ammonium bicarbonate, and 10% of the sample was saved for immunoblotting analysis. Bound proteins were removed from the magnetic beads with 100 μl of Laemmli SDS-sample buffer saturated with biotin at 98 °C for 10 min. BioID samples and controls were analyzed by mass spectrometry in at least three biological replicates^36^.

### Mass spectrometry

Samples were reconstituted in 50 mM ammonium bicarbonate with 10 mM TCEP [Tris(2-carboxyethyl)phosphine hydrochloride; Thermo Fisher Scientific], and vortexed for 1 h at 37 °C. Chloroacetamide (Sigma-Aldrich) was added for alkylation to a final concentration of 55 mM. Samples were vortexed for another hour at 37 °C. One microgram of trypsin was added, and digestion was performed for 8 h at 37 °C. Samples were dried down and solubilized in 5% ACN-0.2% formic acid (FA). The samples were loaded on a 1.5 ul pre-column (Optimize Technologies, Oregon City, OR). Peptides were separated on a home-made reversed-phase column (150-μm i.d. by 200 mm) with a 56-min gradient from 10 to 30% ACN-0.2% FA and a 600-nl/min flow rate on an Easy nLC-1000 connected to a Q-Exactive HF (Thermo Fisher Scientific, San Jose, CA). Each full MS spectrum acquired at a resolution of 60,000 was followed by tandem-MS (MS–MS) spectra acquisition on the 15 most abundant multiply charged precursor ions. Tandem-MS experiments were performed using higher energy collision dissociation (HCD) at a collision energy of 27%. The data were processed using PEAKS X (Bioinformatics Solutions, Waterloo, ON) and a Uniprot human database (20349 entries). Mass tolerances on precursor and fragment ions were 10 ppm and 0.01 Da, respectively. Fixed modification was carbamidomethyl (C). Variable selected posttranslational modifications were oxidation (M), deamidation (NQ), phosphorylation (STY). The data were visualized with Scaffold 4.3.0 (protein threshold, 99%, with at least 2 peptides identified and a false-discovery rate [FDR] of 1% for peptides).

### Immunoblotting and immunoprecipitation

Immunoprecipitation and blotting was performed as previously described^36^. Cells were lysed in RIPA lysis buffer (50 mM Tris–HCl, pH 8, 0.15 M NaCl, 0.1% SDS, 1% NP-40, 1% sodium deoxycholate, 50 mM NaF, 5 mM orthovanadate, 1 mM DTT) supplemented with proteinase inhibitor cocktail (Sigma # 11836170001). Total proteins were separated by SDS-PAGE and transferred to Nitrocellulose membrane (Bio-rad # 1620115). The primary antibodies and secondary horseradish peroxidase antibody were used. The primary antibodies were used as follows: aPKCι 1/1000 (BD Transduction #610175), PKCzeta 1/1000 (Cell signaling #9368S), Streptavidin 1/10000 (Jackson IR through #Cedarlane), α-Tubulin 1/5000 (Sigma #T9026), PAR6B 1/1000 (Santa Cruz #sc-67393), mCherry 1/1000 (Abcam #ab167453), GAPDH 1/1000 (Novus Biologicals #NB300-322), myc 1/1000 (Origene # TA150121), PARD3A 1/1000 (Sigma #07-330), PARD3A 1/1000 (Millipore #07-330), PARD3B 1/1000 (Santa Cruz #sc-398761), and PTPN14 1/1000 (R&D Systems #MAB4458), V5 1/5000 (Thermo Fisher Scientific # R960-25). The proteins were detected by enhanced chemiluminescence method. Bands were visualized using SuperSignal West Pico Chemiluminescent Substrate (Bio-rad #1705061) or Clarity Max Western ECL substrate (Bio-rad # 1705062S) and visualized on UltraCruz radiographic film (Santa Cruz # sc-201696). For immunoprecipitation, cells were washed twice with ice-cold PBS and then lysed in NP40 buffer (150 mM NaCl, 1% NP-40, 50 mM Tris–HCl, pH 8.0) containing a protease inhibitor cocktail. Lysates were precleared with MagnaBeads (Thermo Fisher Scientific #12321D) and then incubated with 2 μg of antibody or isotype control overnight at 4 °C. Antibodies were captured with MagnaBeads and washed three times with NP40 buffer^36^.

### Immunostaining and imaging

Immunostaining and imaging were performed as previously described^36^. Cells from three-dimensional cultures were fixed with 2% paraformaldehyde/PBS for 10 min, permeabilized in 0.5% Triton X-100/10% Goat serum/10% fish gelatin/PBS for 1 h and incubated overnight in primary antibodies. Primary antibodies used were as follows: PAR6B 1/200 (Santa Cruz #sc-67393), aPKCι 1/100 (BD Transduction #610175), PARD3B 1/100 (Santa Cruz #sc-398761), myc 1/100 (Origene # TA150121), GFP 1/500 (Abcam #ab13970), PTPN14 1/100 (R&D Systems #MAB4458), E-cadherin 1/200 (Cell Signaling #3195S), V5 1/200 (Thermo Fisher Scientific # R960-25), β1-integrin 1/200 (Abcam #ab30394), ZO-1 1/100 (Cell Signaling #8193S), YAP 1/100 (Cell Signaling #14074) and Phalloidin 1/100 (Invitrogen #A34055). The secondary antibodies conjugated to Alexa488, Alexa546, Alexa647, and Cy3 (Jackson ImmunoResearch Laboratories) were used at 1:750. DNA was detected with Hoechst dye 33258. Confocal imaging was performed using a LSM700 microscope from Zeiss with 20X/0.8NA or 40X/1.4NA objective lenses and processed using FIJI/ImageJ^37^ (version 1.53c, https://imagej.net/software/fiji/)^36^.

### Statistical analysis

Unpaired student’s t-tests were used to comparison two independent means using Excel and GraphPad Prism 6. Images are representative of three or more independent replicates.

## Results

### Suspension culture of Caco-2 cells supports lumen formation

**Figure 1:**
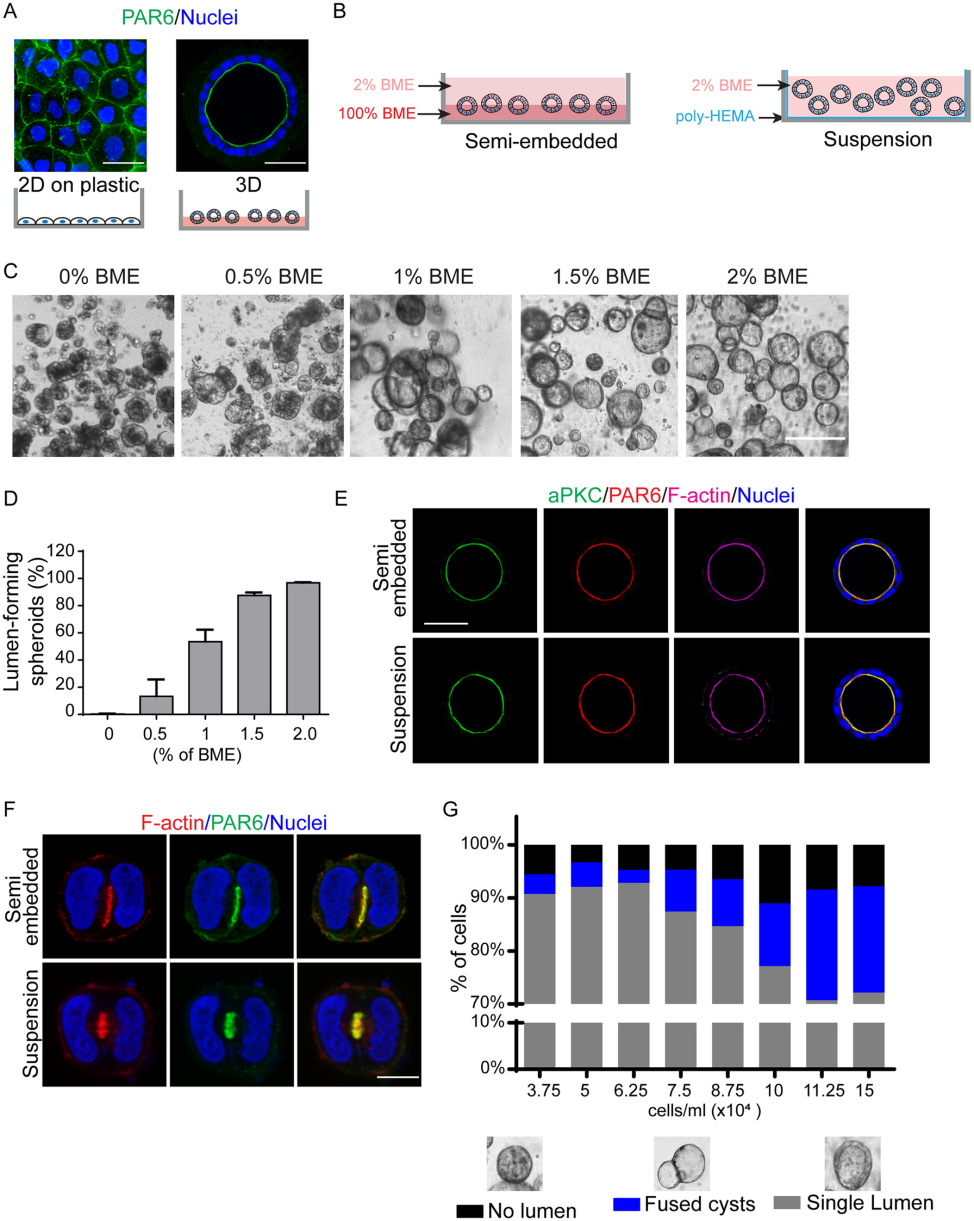
Suspension culture of Caco-2 cells supports lumen formation. (**A**) Confocal images for PAR6B (green) in 2D and 3D Caco-2 cells. (**B**) Schematic showing 3D organotypic cell culture model for semi-embedded and suspension Caco-2 cells. (**C**) Brightfield images showing the phenotype of 3D organotypic suspension Caco-2 cells in different percentage (0–2%) of BME. (**D**) Quantification of the percentage of lumen-forming in different percentage (0–2%) of BME. (**E**) Confocal images were captured of cells cultured in 3D semi-embedded or suspension cultures and immunostained for aPKC (green), PAR6 (red), and F-actin (magenta). (**F**) Confocal images of 2-cell structures grown as 3D semi-embedded or suspension cultures and immunostained for PAR6 (green) and F-actin (red). (**G**) The quantification of Caco-2 cells phenotypes (no lumen, fused cysts, single prominent cyst) following culture at various cell densities (3.75–15 × 10^4^ cells/ml) in suspension cell culture. Cells were seeded in 8-well ibidi slides. Scale Bars: A, E, 50 µm; C, 100 µm; F, 10 µm.

To identify potential novel regulators of lumen formation, we chose Caco-2 cells because they efficiently form lumen in 3D culture^38^. Culture of cells in 3D environments for lumen formation typically involves embedding cells in solid basement membrane extract (BME) or culturing cells on top of a solid layer of BME with 2% soluble BME in the culture medium (Fig. 1A). While these formats efficiently generate 3D structures with a lumen, there are limitations for their use in proteomic analysis. First, embedding cells within or on top of solid BME is difficult to scale up and is costly to obtain sufficient cells for proteomic screening. Second, cells need to be extracted from the solid BME gels and processed prior to proteomic analysis, which can lead to sample loss. Therefore, we sought to develop a suspension culture format to culture 3D Caco-2 cysts that would alleviate these challenges.

For suspension cultures, we generated non-adhesion dishes by coating standard tissue culture dishes with polyHEMA, an inert biopolymer that prevents cell adhesion to the plastic surface (Fig. 1B). To determine if cells could grow into 3D structures with a central lumen in suspension culture, we seeded single cell suspensions in the presence 0–2% BME and cultured them for 9 days. Cells showed a BME concentration-dependent effect on lumen formation that was maximal (> 95%) in 2% BME (Fig. 1C,D). Although cysts grown in 1.5% BME were able to form a lumen with > 80% efficiency, they tended to fuse together, whereas this was minimal in 2% BME. Therefore, 2% BME represented the optimal concentration for lumen formation in suspension Caco-2 cells. Immunostaining for apical markers (aPKC, PAR6B, F-actin) confirmed that cell polarity and epithelial architecture was indistinguishable between Caco-2 cells cultured in semi-embedded or non-adherent conditions (Fig. 1E). In semi-embedded cultures, Caco-2 and MDCK cells establish polarity through the formation of a pre-apical patch at the 2-cell stage that subsequently expands the apical membrane to form a lumen, whereas other cells (e.g. MCF10A) establish polarity through apoptosis-mediated cavitation^7,38,39^. Staining of polarity markers (F-actin and PAR6B) in 2-cell structures from suspension culture shows the presence of a pre-apical patch (Fig. 1F), indicating that de novo lumen formation occurs similarly for Caco-2 cells in semi-embedded and suspension cultures. An advantage of non-adherent cultures is the potential to scale cell production. To this end, we examined lumen formation efficiency at a range of cell concentrations (3.75–15 × 10^4^ cells/ml). Up to 6.25 × 10^4^ cells/ml, > 90% of cells generated 3D structures with a single lumen, similar to semi-embedded cysts, whereas above this, the efficiency deteriorated with increased proportion of fused cysts or cysts with no lumen (Fig. 1G). For all subsequent experiments, cells were cultured at or below to 6.25 × 10^4^ cells/ml.

### Validation of BirA*-PAR6B expression and localization

**Figure 2:**
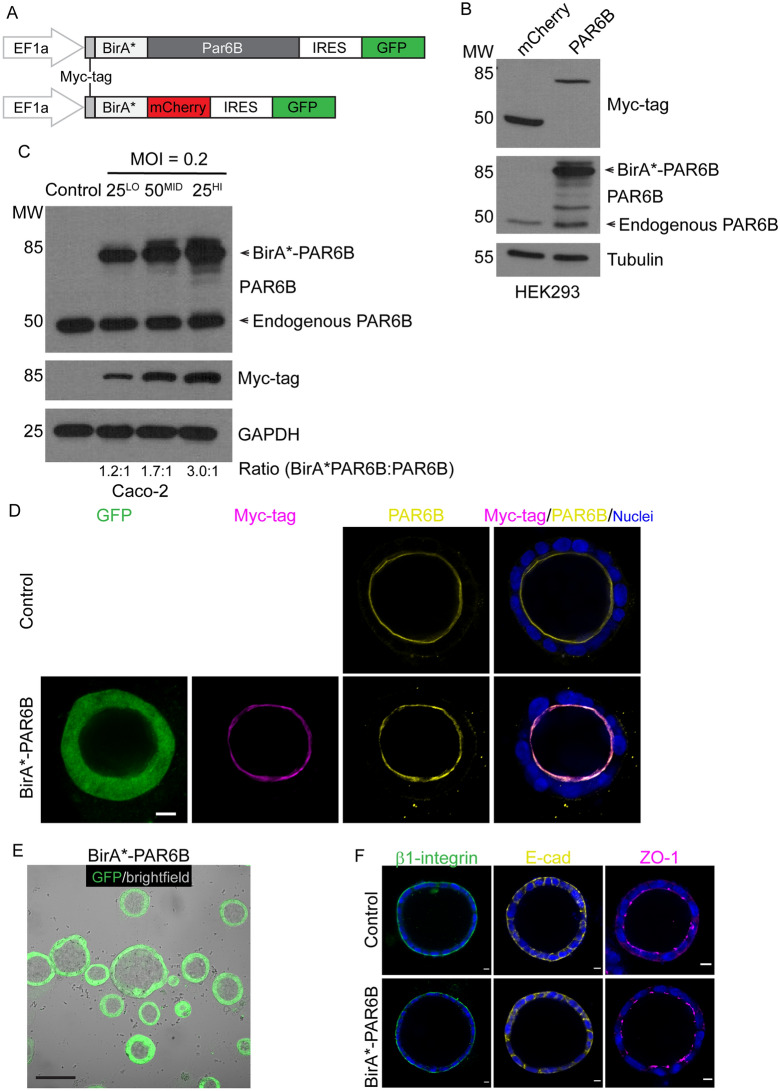
Validation of BirA*-PAR6B expression and localization. (**A**) Schematic showing lentiviral constructs for the expression of BirA*PAR6B and BirA*-mCherry. Expression was driven by an EF1α promoter, and an internal ribosomal entry site (IRES) directs expression of GFP. (**B**) Myc-tagged BirA*-mCherry (control) and BirA*-PAR6B expression were confirmed in HEK293 cells by western blot analysis. (**C**) To provide consistent expression levels, Caco-2 cells were infected with lentiviral constructs shown in (A) at a low multiplicity of infection (MOI = 0.2) to ensure single site integration. Three levels of expression were isolated by FACS of the coupled GFP marker: lowest 25%, the middle 50%, and the highest 25%. The expression of BirA*-PAR6B was determined by western blot with PAR6B antibodies. The ratio of BirA*-PAR6B: endogenous PAR6B is denoted at the bottom of the blot. (**D**) Confocal images were captured for control (uninfected) and BirA*-PAR6B-expressing 3D Caco-2 cells, immunostained for myc-tag (magenta) and PAR6B (yellow), to demonstrate that BirA*-PAR6B localizes to the apical membrane. GFP was visualized by direct fluorescence. (**E**) Widefield images (brightfield and GFP fluorescence) were captured and overlaid, demonstrating that BirA*-PAR6B-expressing 3D Caco-2 cells form a single prominent lumen. (**F**) Confocal images were captured for control (uninfected) and BirA*-PAR6B-expressing 3D Caco-2 cells, immunostained for β1-integrin (green), E-cad (yellow), and ZO1 (magenta), to demonstrate that basal, lateral, and apical markers are properly localized. Scale Bars: D, F, 10 µm; E, 100 µm.

To identify proteins that may be involved in lumen formation, we chose to use proximity biotinylation (BioID) with PAR6B, an established regulator of lumen formation that associates with both the Par- and Crumbs-complexes^6,14^. We used lentiviral plasmids expressing a myc-tag between BirA* and the fusion protein, as well as an IRES-GFP cassette to identify transduced cells (Fig. 2A). We initially confirmed expression of BirA*PAR6B and a BirA*-mCherry as a control in HEK293 cells, which produced fusion proteins at the expected sizes (BirA*-PARD6B, 85 kDa; BirA*-mCherry, 50 kDa) (Fig. 2B).

We next used lentivirus to generate stable expression of BirA*-PAR6B and BirA*-mCherry in Caco-2 cells. To obtain exogenous expression levels of BirA*-fused PAR6B similar to endogenous levels of PAR6B, we used a low dose of lentivirus virus (Multiplicity of Infection (MOI = 0.2) and used fluorescent activated cell sorting (FACS) to split cells into three groups based on GFP expression (lowest 25%, middle 50%, highest 25%). The expression of BirA*-PAR6B was evaluated by western blot, and it was determined that it was expressed similar to endogenous PAR6B levels in the cells with lowest GFP-expression (25%, Fig. 2C). We confirmed that at this expression level, BirA*-PAR6B localized to the apical membrane, like endogenous PAR6B, and did not disrupt lumen formation or β-integrin, E-cadherin, or ZO1 localization (Fig. 2D–F), indicating that polarity is intact.

### Identification of PAR6B proximity proteins in polarized Caco-2 cells

**Figure 3:**
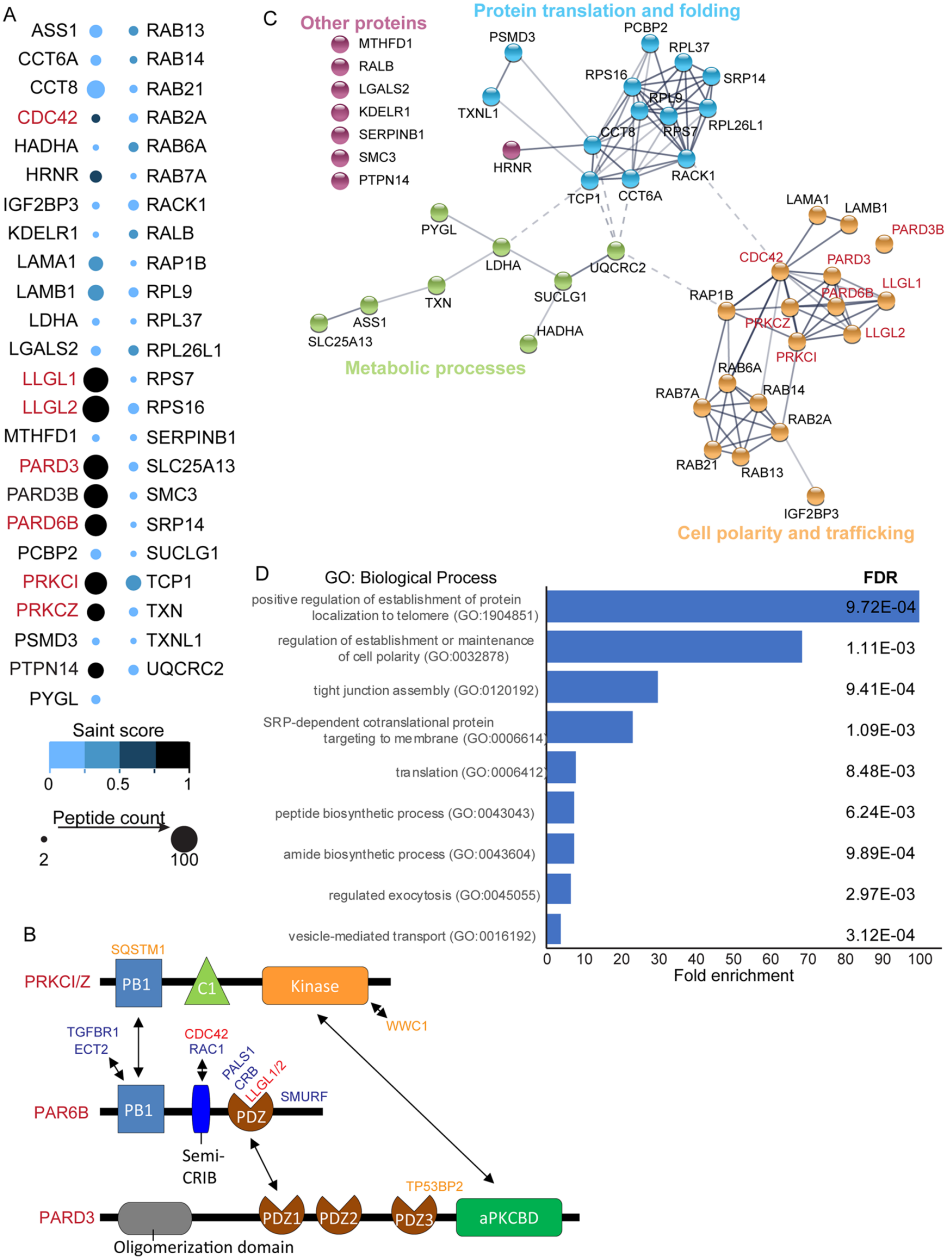
Identification of PAR6B proximity proteins in polarized Caco-2 cysts. (**A**) The SAINT score (dot color) and peptide counts (dot sizes) for PAR6B-proximity proteins identified in 3D BirA*-PAR6B-expressing Caco-2 cells are shown. Proteins known to associate with the apical Par complex are shown in red. (**B**) Diagram shows known interaction domains and interactors for Par complex (aPKC, PAR6B, PARD3). Proteins in red are known interactors for PAR6B and were identified in BirA*-PAR6B mass spectrometry data. Proteins in blue are known interactors for PAR6B and were not identified in BirA*-PAR6B mass spectrometry data. Proteins in orange represent interactors of aPKC or PARD3 that are not known to interact with PAR6B that were identified as proximal to BirA*-PAR6B. (**C**) Diagram showing connections between PAR6B-proximal proteins based on data from the STRING database (version v11.5, https://string-db.org/). Line darkness indicates the strength of the predicted relationship between the proteins. (**D**) Graph shows fold enrichment of different biological processes of for PAR6B proximal proteins based on the Gene Ontology database (version, 10.5281/zenodo.1205166)^44–46^. FDR: False Discovery Rates.

To identify apical proteins proximal to PAR6B, we grew cells for 8 days in suspension culture, a time when lumen are established. At this time, we added biotin for 48 h, then lysed cells and pulled-down biotinylated proteins using streptavidin beads. In parallel, we performed experiments on Caco-2 cells cultured on plastic tissue culture dishes in a two-dimensional (2D) format to determine if unique proteins could be identified in Caco-2 cells that form a lumen (3D) and those that polarize in the absence of lumen formation (2D). From triplicate experiments, 8645 peptides from 526 proteins were identified in 3D samples, whereas 13,329 peptides from 600 proteins were identified in 2D samples. We filtered the list of PAR6B-proximity proteins using the following criteria: (1) peptides were detected in at least two of the three replicates; (2) the average peptide count was at least twice the count in the control; (3) the peptide count was < 10 for each of the control replicates. This filtering process resulted in 47 proteins in 3D samples and 34 proteins in 2D (Fig. 3A and Supplementary Fig. S1A). This filter is justified, since it includes known PAR6B-associated proteins, whereas more stringent cut-offs excluded some known interactions (Fig. 3A,B). High confidence hits (Saint Score > 0.75) included polarity proteins known to directly associate with PARD6B (LLGL1, LLGL2, PARD3, PRKCI (aPKCi), PRKCZ (aPKCz), and CDC42), as well PARD3B^12^. We also identified PTPN14 and HRNR as high-confidence hits with unknown roles in cell polarity or lumen formation. As expected, known PAR6B-associated proteins were also identified as high-confidence hits from cells grown in 2D (Supplementary Fig. S1A). Additional proteins known to associate with the Par complex (WWC1/Kibra, TP53BP2/ASPP2, SQSTM1/p62) were also identified as lower confidence interactions (Saint Score < 0.75; (Supplementary Fig. S1A,B)^40,41^. Strikingly, we did not observe proteins from the Crumbs complex in proximity to PAR6B in either 2D or 3D experiments, despite reported data supporting PAR6B as a component of both the Par and Crumbs complexes^42,43^. This could result from the presence of peptides below our detection limit or less efficient biotinylation of these proteins due to conformation of the complex and proximity of these targets to BirA*.

To further explore relationships between PAR6B proximity proteins, we used STRING protein–protein interaction networks as well as Gene Ontology for candidates from 2D and 3D samples (Fig. 3C,D and Supplementary Fig. S1B,C). This identified three clusters related to (1) Apical basal polarity and trafficking, (2) protein translation and folding, and (3) metabolic processes. Of the PAR6B-proximal proteins identified in 3D and 2D screens, 40 were common, 19 specific to 3D samples and 10 specific to 2D samples (Supplemental Fig. S2A). Furthermore, for two proteins (PARD3B and RALB), peptide spectra were identified in both 2D and 3D samples, but they were enriched > fivefold in the 3D samples, indicating some differences in proximity networks between 2 and 3D samples (Supplemental Fig. S2B). To determine if differences in observed peptide spectral counts between 2D and 3D could result from differences in gene expression, we compared 2D and 3D using RNA-seq. However, mRNA expression was not consistently elevated for the proteins that were exclusive or enriched to the 3D samples (Supplemental Fig. S2C).

### PARD3B and other PARD6B-proximity proteins are required for lumen formation

**Figure 4:**
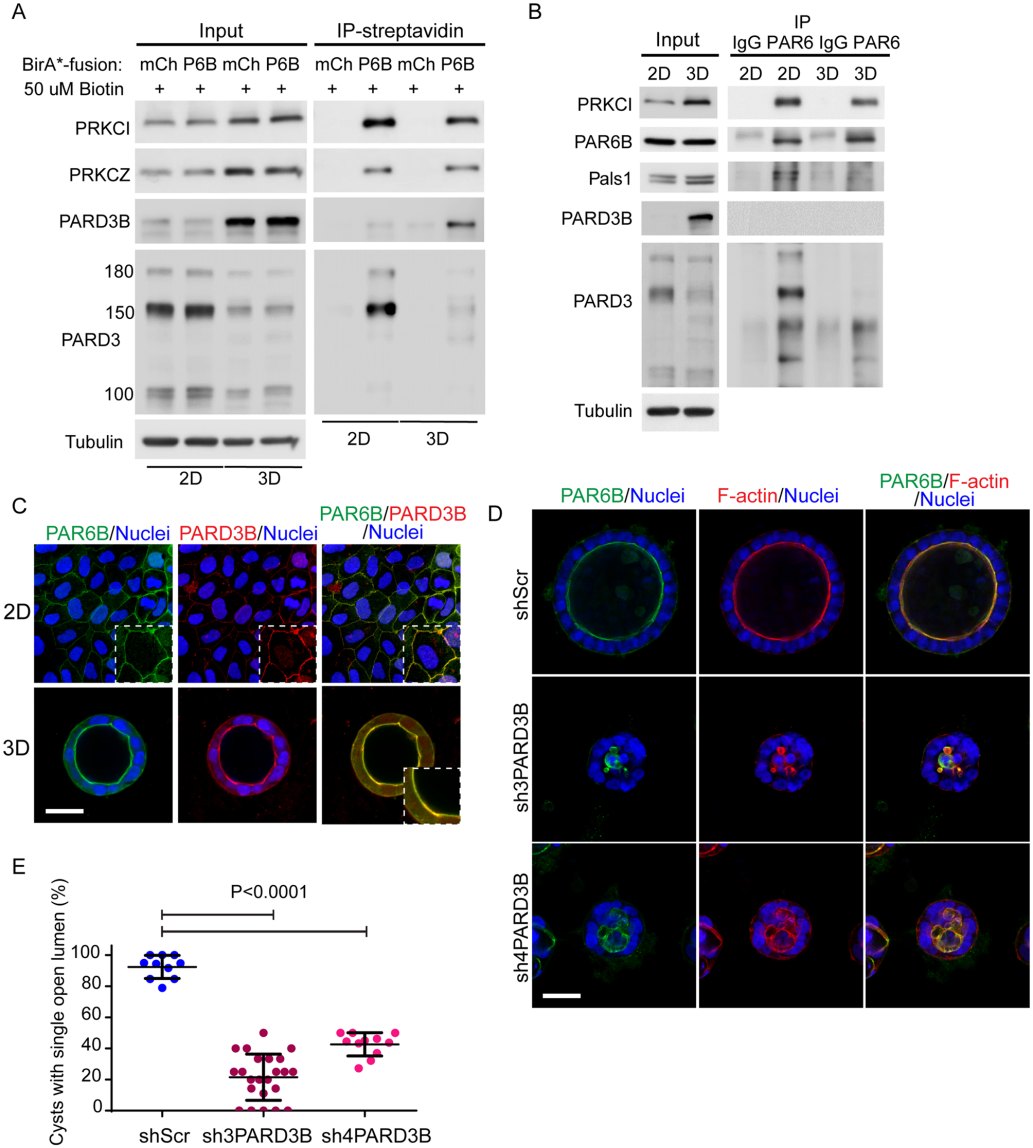
PARD3B associates with a Par-complex and is required for lumen formation. (**A**) Immunoprecipitation was performed with streptavidin in 2D and 3D BirA*-mCherry and BirA*-PAR6B-expressing Caco-2 cells. (**B**) Immunoprecipitation was performed with anti-PAR6B in 2D and 3D Caco-2 cells. (**C**) Confocal images were captured for 2D (top panel) and 3D (bottom panel) BirA*-PAR6B-expressing Caco-2 cells immunostained for PAR6B (green) and PARD3B (red). (**D**) Confocal images were captured for 3D shScr, sh3PARD3B, and sh4PARD3B knock-down Caco-2 spheroids in semi-embedded gels and immunostained for PAR6B (green) and F-actin (red). (**E**) Quantification of the percentage of cysts with single open lumen in shScr, sh3PARD3B, and sh4PARD3B knock-down Caco-2 spheroids. Scale Bars: C, 30 µm; D, 50 µm.

PARD3B has been shown not to associate with the Par-complex^11,12^, so we were surprised to find it in proximity to PAR6B in our experiments. Moreover, we were intrigued that it was enriched in 3D samples compared to cells cultured in 2D on plastic. We first confirmed the results obtained from mass spectrometry analysis that PARD3B is enriched in streptavidin pull-downs from Caco-2 cells expressing BirA*-PAR6B in 3D versus 2D/plastic cultures (Fig. 4A). Moreover, PARD3 was a less abundant proximity partner for PAR6B in 3D versus 2D, whereas PRKCI and PRKCZ were equivalent as detected by immunoblot, suggesting there may be a PAR3 isoform switch between 2D/plastic and 3D cultures. Consistent with this idea, we observed opposite expression profiles between the major splice form of PARD3 (150 kDa) and PARD3B in 2D and 3D Caco-2 cells in whole cell lysates (Fig. 4A,B). Expression of PARD3 and PARD3B mRNA were not different between 2D/plastic and 3D cultures, nor was their expression changed in 2D cultures following treatment with the proteasome inhibitor MG132, indicating that the difference in expression of Par3 isoforms between 2D/plastic and 3D environments is not controlled by transcription or proteasomal degradation (Supplemental Fig. 3A). We were unable to detect an association between endogenous PARD3B and PARD6 or PARD3 and PARD3B by co-immunoprecipitation (Fig. 4B, Supplemental Fig. S3B), which is consistent with previous results (Kohjima et al., 2002) and suggests that association of PARD3B with the Par-complex may not be stable under tested immunoprecipitation conditions. Finally, we examined the localization of PARD3B in both 2D/plastic and 3D cultures, which revealed colocalization with PAR6B at junctions (2D) and the apical membrane (3D) (Fig. 4C). Taken together, these data support an association between PARD3B and PAR6B, although more thorough studies will need to be performed to further understand a PARD3B-containing polarity complex.

To examine the function of PARD3B in lumen formation, we evaluated two independent shRNA targeting PARD3B in Caco-2 cells grown in semi-embedded 3D culture. Whereas control cells generated cysts with a single prominent lumen, cells expressing shRNA to PARD3B formed cysts with small multiple lumen (Fig. 4D,E), reminiscent of the multi-lumen phenotype observed when PARD3, PRKCI, or PARD6B is depleted in 3D culture^14,47^. A similar phenotype was obtained following knockdown of PARD3B in suspension culture, further supporting that cells grown in suspension (Supplementary Fig. 3D) are functionally comparable to those grown in standard semi-embedded gels. In 2D/plastic cultures, expression of PARD3B shRNA did not impair stable cell polarization or junction formation, as determined by immunostaining for PARD6B and F-actin (Supplemental Fig. S3C). Collectively these results demonstrate that PARD3B is required for normal lumen formation and support a role of PARD3B as a part of a polarity-complex in this process.

To validate whether additional PAR6B-proximity proteins identified in our BioID screen were involved in lumen formation, we examined HRNR and RALB, which to our knowledge have not previously been implicated in lumen formation or apical-basal polarity. RALB is a small GTPase that is associated with a variety of cellular processes, including regulation of endocytosis, exocyst function, cell migration, and transcription^48^. To investigate if RALB is involved in lumen formation, we examined 3D Caco-2 cells expressing control shRNA (shScr) or one of two-independent shRNA directed to RALB (sh1-RALB and sh5-RALB). Whereas cells formed a single layer surrounding the lumen in control cysts, cysts expressing RALB-shRNA displayed collapsed disorganized lumen with regions of multilayered cells (Supplementary Fig. S4A,B). Despite RALB-shRNA-expressing cysts having a smaller size, the number of cells/cyst cross section was similar to the control (Supplementary Fig. S4C), indicating that the smaller size was due to the absence of a lumenal space. In 2D/plastic cultures, RALB-depleted cells had no obvious alterations in cell polarity or epithelial organization (Supplemental Fig. S4D).

HRNR (Hornerin) is a S100 family member that is required for epidermal barrier formation and carcinoma progression^49,50^. We investigated a potential role for HRNR in lumen formation and epithelial organization. In 3D cysts, expression of two independent HRNR-shRNA resulted in multiple microlumen and disorganized structures with small, condensed nuclei (Supplementary Fig. 5A–C). Moreover, 2D/plastic cultures also showed severe epithelial disorganization, with large cells with irregular nuclear size and disrupted cortical PAR6B and F-actin (Supplemental Figs. S5D–F). These data demonstrate that the proximity screen identified functionally-relevant regulators of lumen formation.

### PTPN14 associates with the Par complex and is controls apical fidelity

**Figure 5:**
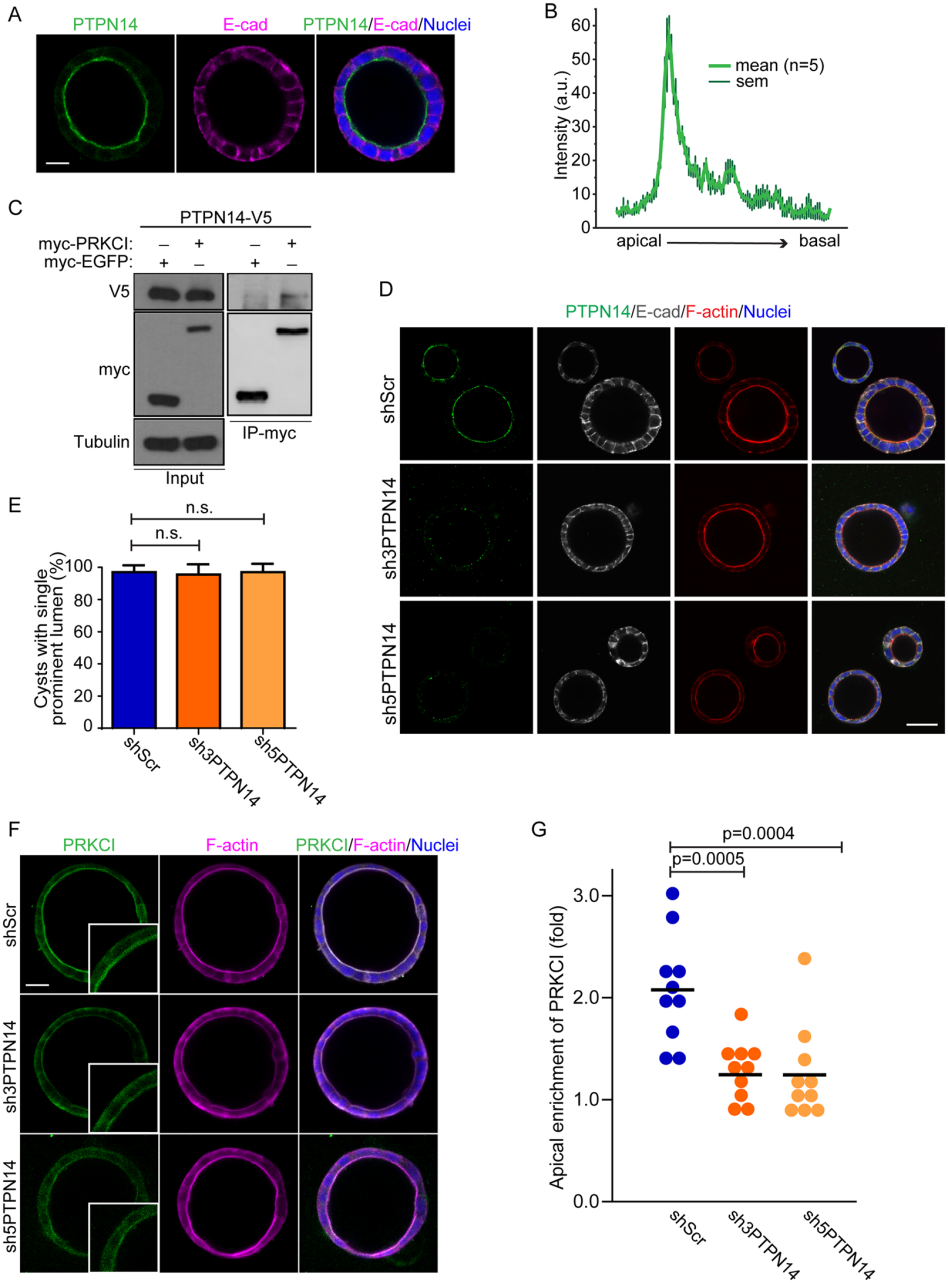
PTPN14 associates with the Par complex and controls apical fidelity. (**A**) Confocal images were captured for polarized Caco-2 cysts immunostained for PTPN14 (green) and E-cad (magenta), showing that PTPN14 localizes to the apical membrane. (**B**) Line tracing shows PTPN14 fluorescent intensity from apical to basal membrane of polarized Caco-2 cysts. (**C**) Co-immunoprecipitation of PTPN14-V5 and myc-EGFP or myc-PRKCI was performed with anti-myc following transient transfection in HEK293 cells. The presence of PTPN14 in immunoprecipitates was determined by western blot analysis using anti-V5. (**D**) Confocal images were captured for shScr, sh3- and sh5-PTPN14 knock-down Caco-2 cysts immunostained for PTPN14 (green), E-cad (grey), and F-actin (red). (**E**) Quantification of the percentage of cysts with single prominent lumen in shScr, sh3-PTPN14 and sh5-PTPN14 knock-down Caco-2 cysts. (**F**) Confocal images for PRKCI (green) and F-actin (magenta) show the phenotype of shScr, sh3-PTPN14 and sh5-PTPN14 knock-down Caco-2 cysts. (**G**) Quantification of the fold change of apical enrichment of PRKCI in shScr, sh3-PTPN14 and sh5-PTPN14 knock-down Caco-2 cysts. Scale Bars: A, 20 µm; D, 50 µm; F, 30 µm.

PTPN14 is a cytosolic non-receptor protein tyrosine phosphatase (PTP) that is highly expressed in multiple epithelial tissues^51,52^. PTPN14 forms a complex with KIBRA and LATS (large tumor suppressor) that regulates the Hippo signaling pathway by dephosphorylating Yap to prevent nuclear localization and transcriptional co-activator activity^53–55^. KIBRA is a scaffold protein with WW, C2-like, and aPKC-binding domains, and it can be phosphorylated by aPKC^56^. We therefore wondered if PTPN14 may be part of an apical complex that regulates apical-basal polarity and lumen formation. To verify this, we first examined the localization of endogenous PTPN14 or exogenous V5-tagged PTPN14 (PTPN14-V5), which colocalized with Par6 and F-actin at the apical surface (Fig. 5A,B, and Supplemental Fig. 6A). To confirm the interaction between PTPN14 and the Par-complex, we co-immunoprecipitated PTPN14-V5 and myc-tagged PRKCI and PAR6B in HEK293 cells (Fig. 5C, Supplementary Fig. 6B). Given that we found it to associate with an apical polarity proteins, we further investigated a role for PTPN14 in apical-basal polarity by depleting it using two-independent shRNA (sh3-PTPN14 and sh5-PTPN14) (Supplemental Fig. 6C). However, we did not observe any obvious defects in the overall cyst morphology or lumen formation (Fig. 5D,E). However, we noted that apical enrichment of PRKCI and PAR6B were moderately reduced in PTPN14-deficient cells, whereas localization of basolateral marker E-cadherin appeared unaffected (Fig. 5D,F,G, Supplementary Fig. 6D,E). Collectively, these results suggest that PTPN14 is required for apical polarity robustness, but this does not affect lumen formation in Caco-2 cysts.

**Figure 6:**
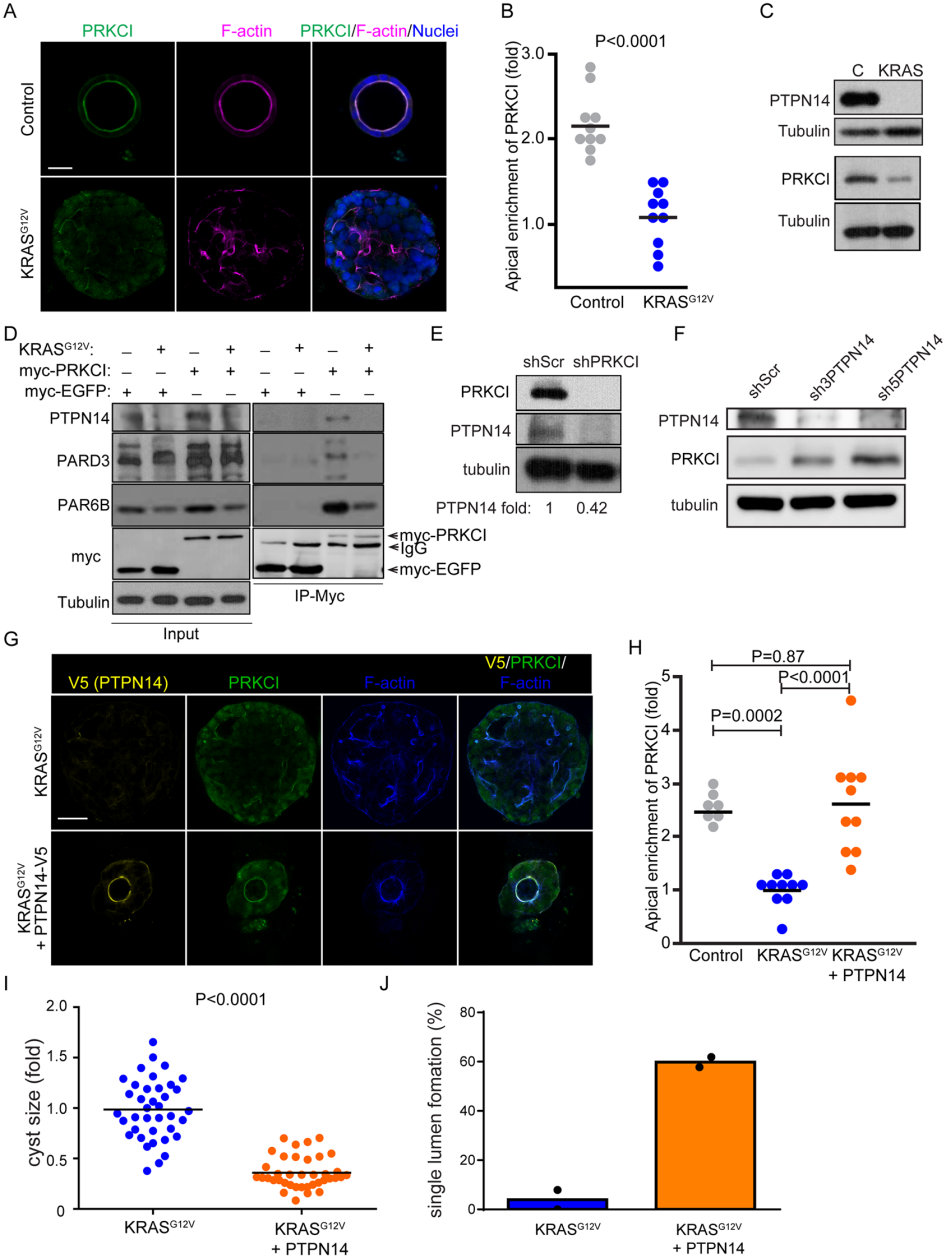
PTPN14 suppresses KRAS^G12V^-induced transformation in Caco-2 cells. (**A**) Confocal images were captured for control and KRAS^G12V^-transformed Caco-2 spheroids immunostained for PRKCI (green) and F-actin (magenta). (**B**) Quantification of the fold change of apical enrichment of PRKCI in control and KRAS^G12V^-transformed Caco-2 cysts. (**C**) Western blot showing PRKCI and PTPN14 protein expression in control and KRAS^G12V^-transformed Caco-2 spheroid lysates. (**D**) Immunoprecipitation of myc-EGFP or myc-PRKCI was performed with anti-myc as control and KRAS^G12V^-transformed Caco-2 cysts. (**E**) Western blot was performed with anti-PRKCI and anti-PTPN14 in PRKCI knockdown Caco-2 cell lysates. (**F**) Western blot probed with anti-PRKCI and anti-PTPN14 antibodies in shScr, sh3- and sh5-PTPN14 knock-down Caco-2 cell lysates. PRKCI was up-regulated in PTPN14 knockdown Caco-2 cells. (**G**) Confocal images were captured for KRAS^G12V^-transformed Caco-2 cysts with or without PTPN14 overexpression immunostained for PTPN14-V5 (yellow), PRKCI (green), and F-actin (blue). (**H**) Quantification of the fold change of apical enrichment of PRKCI in control, KRAS^G12V^-transformed Caco-2 cysts, and KRAS^G12V^-transformed Caco-2 cysts with PTPN14 overexpression. (**I**) Quantification of the fold change of cyst size of cultured KRAS^G12V^-transformed Caco-2 cysts with or without PTPN14 overexpression. (**J**) Quantification of the percentage of single lumen formation of cultured KRAS^G12V^-transformed Caco-2 cysts with or without PTPN14 overexpression. Scale Bars: A, 30 µm; G, 100 µm.

### PTPN14 suppresses KRAS^G12V^-induced transformation in Caco-2 cells

Disruption of apical-basal polarity and lumen integrity are features of carcinoma^15^, and KRAS expression in Caco-2 generates cysts with a filled lumen^57^. Moreover, accumulating evidence indicates PTPN14 functions as a tumor suppressor^58^. To further explore the effect of oncogenic KRAS on polarity, we expressed KRAS^G12V^ in Caco-2 cells. KRAS^G12V^-expression generated solid spheres of cells with collapsed microlumen demarcated by F-actin with reduced apical enrichment of PRKCI and undetectable PTPN14 at the apical membrane (Fig. 6A,B, and Supplemental Fig. 7A). Immunoblot analysis of cell lysates indicated that PTPN14 and PRKCI protein were downregulated in KRAS^G12V^-transformed Caco-2 cysts compared to controls, whereas mRNA was unaffected (Fig. 6C, and Supplemental Fig. 7B).

Since polarity was altered by KRAS^G12V^ expression, we investigated whether it affected PAR6B and PARD3 expression and their ability to assemble the Par complex. We observed that PAR6B was also reduced in KRAS^G12V^-expressing samples, consistent with co-stability of PRKCI and PAR6B^14^, whereas PARD3-expression was not affected by KRAS^G12V^ (Fig. 6D). To determine if KRAS^G12V^-expression affected the ability of the Par complex to form, we expressed myc-PRKCI in Caco-2 cells and immunoprecipitated with anti-myc antibodies. In control samples (i.e., without KRAS^G12V^), endogenous PTPN14 co-precipitated with PRKCI, as did PAR6B and PARD3, indicating that PTPN14 is part of a Par-complex. PAR6B was detected in the pull-down of both control and KRAS^G12V^-expressing cells, consistent with their constitutive association (Fig. 6D). The reduced PAR6B band size in the KRAS^G12V^ compared to control pull-down likely reflects lower expression levels of PAR6B in these KRAS^G12V^ cells. In contrast, PARD3 was not detected in the myc-PRKCI pull-down containing KRAS^G12V^ (Fig. 6D), indicating that the Par-complex is disrupted.

Given the association between PRKCI and PTPN14 and the simultaneous down-regulation of both proteins in response to KRAS^G12V^-expression, we wondered whether one may regulate the expression of the other. To understand this potential relationship, we knockdown PRKCI or PTPN14 in Caco-2 cells. Whereas knockdown of PRKCI resulted in a strong reduction in PTPN14-protein, knockdown of PTPN14 caused an up-regulation of PRKCI-expression (Fig. 6E,F). This indicates that PTPN14 associates with PRKCI, and each reciprocally influences the protein expression of the other. Since PTPN14-expression requires PRKCI, it is likely that reduction of PTPN14 in KRAS^G12V^-expressing cells is a consequence of reduced PRKCI expression. However, we cannot exclude that PTPN14 may be affected by KRAS^G12V^ through an alternative mechanism.

To determine if reduced PTPN14 in KRAS^G12V^-expressing cells has a functional consequence for the malignant phenotype (enlarged solid structures with reduced polarity), we enforced expression of V5-tagged PTPN14 in KRAS^G12V^-expressing Caco-2 cells using lentivirus. Strikingly, overexpression of PTPN14 resulted in maintenance of a prominent open lumen, enrichment of PRKCI to the apical membrane, and reduced the size of KRAS^G12V^-expressing Caco-2 cells (Fig. 6G–J). Moreover, in 2D cultures, enforced expression of PTPN14 reduced the growth of KRAS^G12V^-expressing cells (Supplemental Fig. 7C,D). Overall, these findings highlight that disruption of cell polarity by KRAS^G12V^ acts through PTPN14 and highlight its pivotal functions in apical-basal polarity organization during cancer progression.

Previous studies have shown that PTPN14 is a tumor suppressor that regulates Yap/Hippo signaling^54,55,59^, and Yap can be a major effector of mutant KRAS during tumor progression^60,61^. We therefore wondered whether PTPN14 may regulate KRAS^G12V^-induced malignant transformation through YAP. However, consistent with previous reports^62^, we did not observe YAP-translocation to the nucleus in KRAS^G12V^-transformed Caco-2 cells (Supplemental Fig. 8A,B). As a positive control that we could detect oncogene-induced YAP nuclear translocation in Caco-2 cells, we expressed doxycycline-induced SRC^527^ (Supplemental Fig. 8C). Therefore, our data indicate that PTPN14 likely acts as a tumor suppressor through a Yap/Hippo-independent mechanism involving apical-basal polarity in this model of KRAS^G12V^ malignant transformation.

## Discussion

The formation of a lumen is essential to create epithelial tubes and acini during tissue morphogenesis, which is disrupted during cancer progression. De novo lumen formation is a multi-step process that coordinates delivery of apical determinants to the cytokinetic midbody to establish an apical membrane initiation site that matures to an apical membrane patch that opens to form a lumen between cells^6,7^. Although the sequence of these events is well-characterized, the molecular mechanisms that control them are not completely understood. Therefore, there is a need to identify additional regulators of lumen formation and tissue architecture to understand normal tissue development and progression to cancer. To address this, we took advantage of this system to use BioID2 to identify novel proteins potentially involved in cell polarity and lumen formation. We first adapted 3D cultures to optimize lumen formation in a suspension culture format, thereby increasing cell yields for proteomic analysis and show that this can be used to identify known and novel proteins involved in apical-basal polarity and lumen formation. An alternative approach to identify novel genes associated with polarization or lumen formation would be to perform a high-throughput functional screen using shRNA or CRISPR libraries. Limitations to this approach are that the effects observed may be indirectly related to polarity signaling^63,64^. An advantage of a proximity-based screen is that it identifies proteins that are likely part of a complex with a protein of interest, which is validated by a functional assay. Our approach is complementary to a recent study of apical polarity complexes in MDCK cells grown on 2D transwell filters, reporting a comprehensive view of PARD3 and PALS1 polarity complexes in polarized epithelial cells^65^.

To date, the function and binding partners of PARD3B remain unclear. In one study, PARD3B was shown to have differential binding to different PAR6B isoforms^11^. However, another study indicated that PARD3B is incapable of binding to PAR6B or aPKC^12^. Although we did not detect a physical interaction between PAR6B and PARD3B using conventional coimmunoprecipitation experiments, we did find that they were proximal based on BioID and immunostaining experiments. The inability to detect a physical connection between PARD3B and PAR6B could reflect a complex that may not be stable under the same conditions used to isolate PARD3/PAR6B complexes. Moreover, we show that PARD3B localizes to the apical domain and is necessary for lumen formation. Interestingly, PARD3B and PAR6B were recently identified in proximity to PALS1 but not PARD3^65^. Therefore, these data support a model by which PARD3B is a component of the apical PAR6B/PALS1 polarity complex^66^. Interestingly, we found that PARD3B protein was expressed at lower levels in 2D/plastic versus 3D cultures, whereas mRNA expression was similar, suggesting that translation or protein stability may control PARD3B protein levels. However, PARD3B was detected in 2D MDCK cells grown on transwell filters^65^, and whether the differences we detected are due to *bona fide* differences between 2 and 3D Caco-2 cultures or incomplete polarization on 2D/plastic is unclear.

RALB, a member of a subfamily of Ras-related GTPases, binds to its effectors like exocysts once it is activated and to mediate epithelial tight junction formation^67,68^. Moreover, the binding of RALB-exocyst (Exo84) recruits to the midbody of cytokinetic bridge to drive abscission during cytokinesis^69^. This indicates that the association of PAR6B and RALB may involve in early cell polarization or mitotic process. However, the mechanism of how RALB regulate in cell polarity or lumen formation requires further investigation.

Hornerin/HRNR is a S100 protein family member, which are involved in numerous biological functions including inflammatory and immune responses, calcium homeostasis, the dynamics of cytoskeleton constituents, as well as fundamental cellular processes and signaling cascades^70^. HRNR has been studied mostly in the skin epithelium^71–74^ but is also expressed in human breast tissue, mammary epithelial, stromal cells and extracellular matrix^75,76^. We report that knockdown of HRNR impairs lumen formation in 3D spheroid culture and disrupts epithelial organization in 2D culture. Future studies will be necessary to further understand the role HRNR in polarized epithelia and whether defects in lumen architecture reflect a general defect in epithelial organization or a more specific role in lumen formation.

Malignant transformation of epithelia is associated with increased proliferation, loss of apical-basal polarity, and disruption of epithelial organization by stratification or lumen filling^15^. We identified PTPN14 as a PAR6B-proximal protein. We report that PTPN14 localizes to the apical membrane and associates with PAR6B and PRKCI, and collectively, this supports that PTPN14 is part of a Par-complex. The physical link between PAR6B/PRKCI and PTPN14 is currently unknown, however, one possibility is through KIBRA. PTPN14 interacts with KIBRA, which is a cytoplasmic protein and has been shown to regulate a variety of cellular functions including cell growth, apoptosis, directional cell migration, mitotic spindle assembly, and MAPK activation^58^. KIBRA can localize at the apical domain and influence cell polarity by inhibiting aPKC, but is also an aPKC substrate^56,77,78^. Furthermore, studies indicate that KIBRA can interact with Par complex or PTPN14 to activate LATS1 and negatively regulate the YAP in hippo signaling pathway^55,79,80^. This suggests that PTPN14 might be in a complex with KIBRA to regulate cell polarity. Our data show that depletion of PTPN14 decreased the enrichment of PRKCI at the apical membrane, but this did not translate into changes in lumen formation. This could result from residual PTPN14 being sufficient for lumenogenesis, or that PTPN14 is required for optimal PRKCI polarization, but not to establish apical-basal polarity per se.

Previous studies demonstrated a link between apical polarity complexes and HIPPO/Yap signaling^65,81^. Moreover, PTPN14 can function as a tumor suppressor by negatively regulating Yap signaling^58^. This is consistent with our data indicating that PTPN14 acts as a tumor suppressor in KRAS^G12V^-transformed cells. However, we did not observe changes in Yap localization in our Caco-2 experimental model, which is consistent with previous reports that KRAS acts independent of Yap signaling in Caco-2 cells^62^. Therefore, our study indicates that PTPN14 likely has additional Yap-independent tumor suppressor functions by regulating cell polarity.

In summary, the collection of findings highlights that importance of three-dimensional culture model for investigating polarity interactions and contributes to a foundation to further understand the mechanisms underlying polarization and lumen formation in normal and cancer contexts.

## Supplementary Information

Supplementary Information.
