# Supplementary material for: A proximity proteomics screen in three-dimensional spheroid cultures identifies novel regulators of lumen formation
Source: Sci Rep. 2021 Nov 23;11:22807. doi: 10.1038/s41598-021-02178-2 (PMC8610992; doi:10.1038/s41598-021-02178-2)

Wang-Supplementary Figure 1

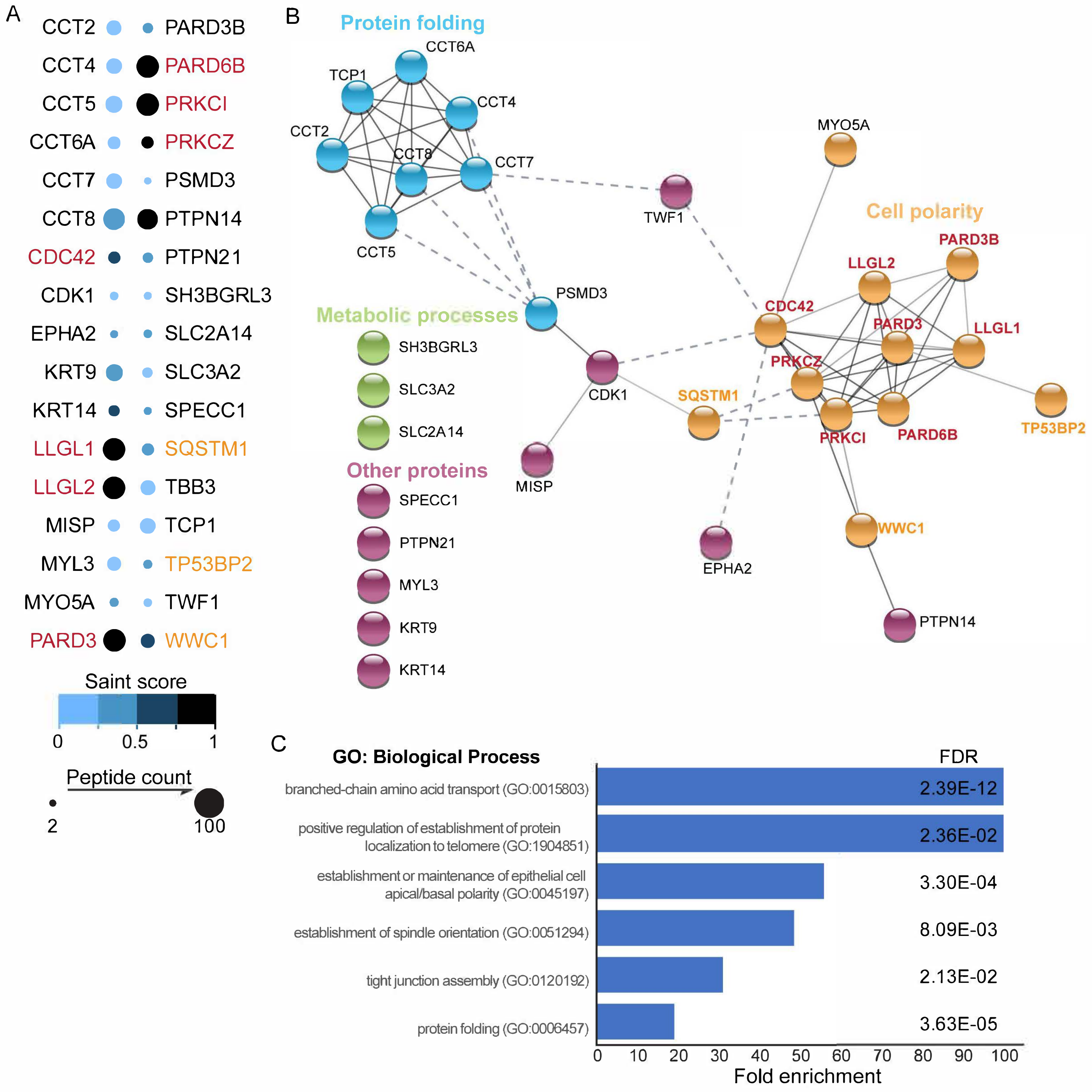

Supplementary Figure 1: Identification of BirA*-PAR6B vicinal proteins in 2D cell culture samples by proteomics analysis. Associated with Figure 3. (A) SAINT scores and peptide counts for PAR6B vicinal proteins identified in 2D BirA*-PAR6B-expressing Caco-2 cells are shown with different color and size. Known PAR6B-associated proteins are shown in red; PARD3 and aPKC-associated proteisn are shown in orange. (B) Connections between PAR6B-vicinal proteins identified in 2D BirA*-PAR6B-expressing Caco-2 based on the STRING database. Line darkness indicates the strength of the predicted relationship between the proteins. (C) The graph shows fold enrichment of different biological processes of BirA*-PAR6B vicinal proteins in 2D samples based on Gene Ontology.

Wang-Supplementary Figure 2

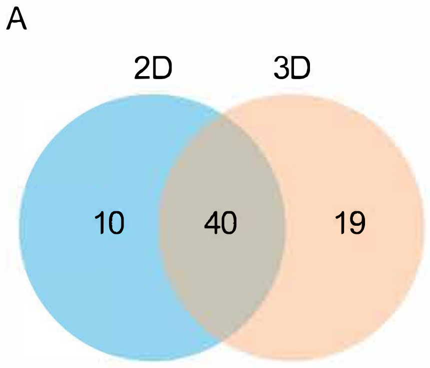

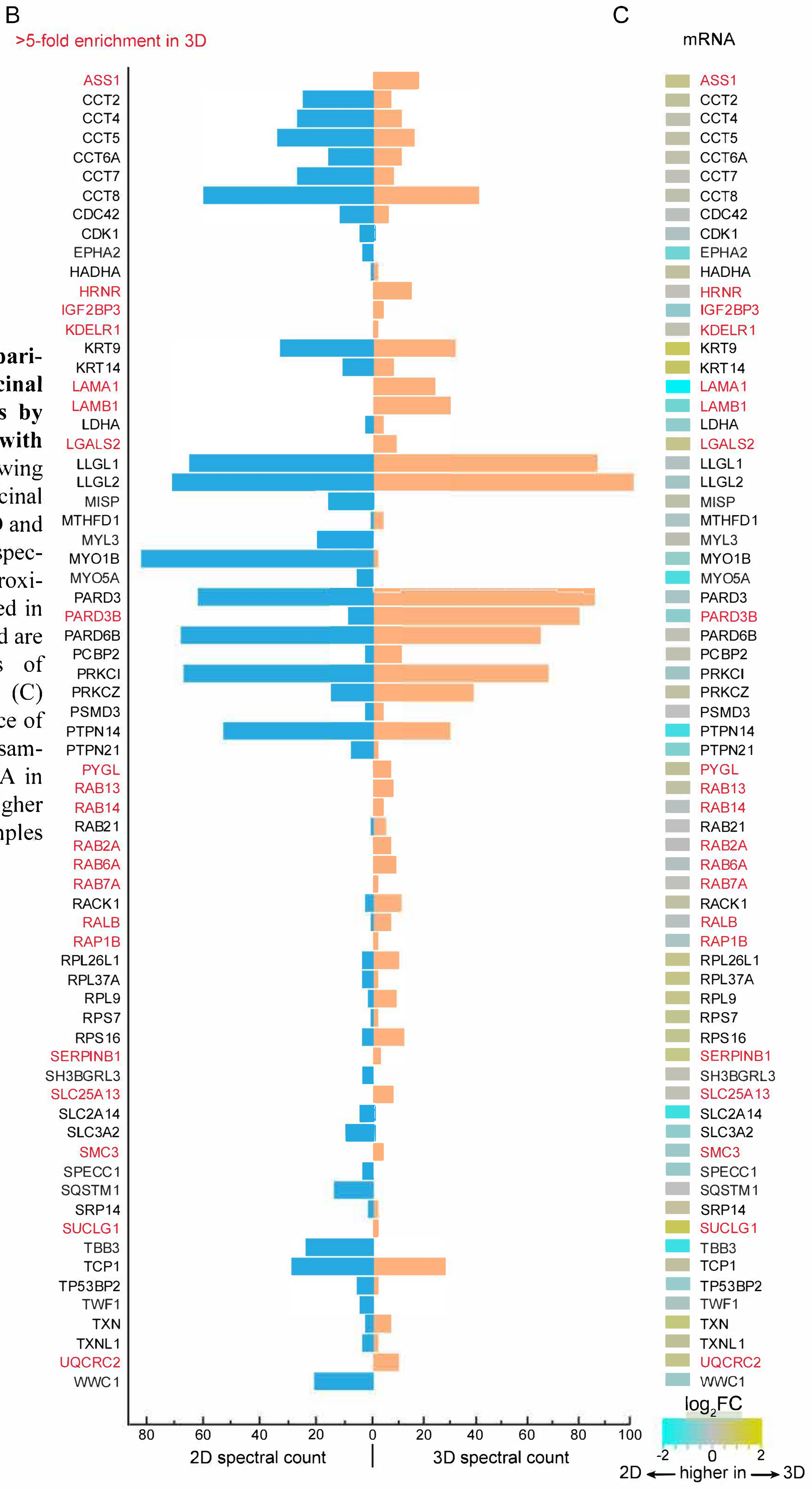

Supplementary Figure 2: Comparison of BirA*-PAR6B vicinal proteins in 2D and 3D screens by proteomics analysis. Associated with Figure 3. (A) Venn diagram showing the number of BirA*-PAR6B vicinal proteins that were identified in 2D and 3D screens. (B) Graph shows the spectral count of the BirA*-PAR6B proximal proteins which were identified in 2D and 3D screens. Proteins in red are more than 5-fold enrichments of peptide spectra in 3D screens. (C) RNA-seq data shows the difference of gene expression in 2D and 3D samples. Higher expression of mRNA in 3D samples are shown in green, higher expression of mRNA in 2D samples are shown in blue.

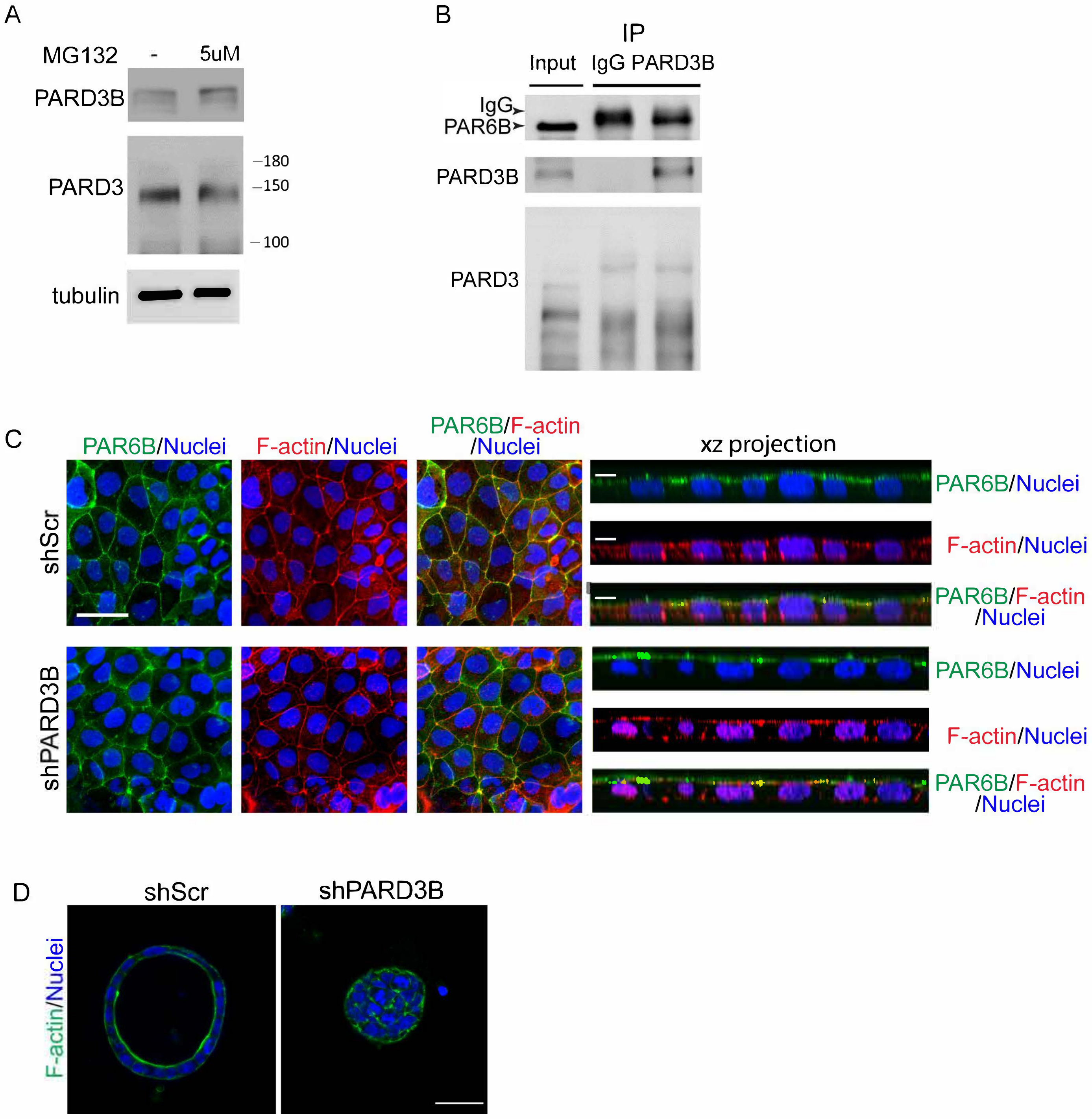

Supplementary Figure 3: PARD3B knockdown does not disrupt the morphology of 2D Caco-2 cells. Associated with Figure 4. (A) Caco-2 cells were treated with solvent or 5uM proteasome inhibitor MG132 for 24 hrs. Protein expression of PARD3B, PARD3, and tubulin was determined by western blot analysis. (B) Immunoprecipitation were performed with anti-PARD3B in 2D Caco-2 cells. (C) Confocal images were captured for 2D PARD3B knockdown Caco-2 cells immunostained for PAR6B (green) and F-actin (red). Orthogonal slices through a field of cells stained for PAR6B (green) and F-actin (red) in 2D PARD3B knockdown Caco-2 cells. (D) Confocal images were captured for suspension 3D shScr, shPARD3B knock-down Caco-2 cysts immunostained for F-actin (green). Scale Bars: C, 50µm (left panel), 10µm (right panel); D, 50µm.

Wang-Supplementary Figure 4

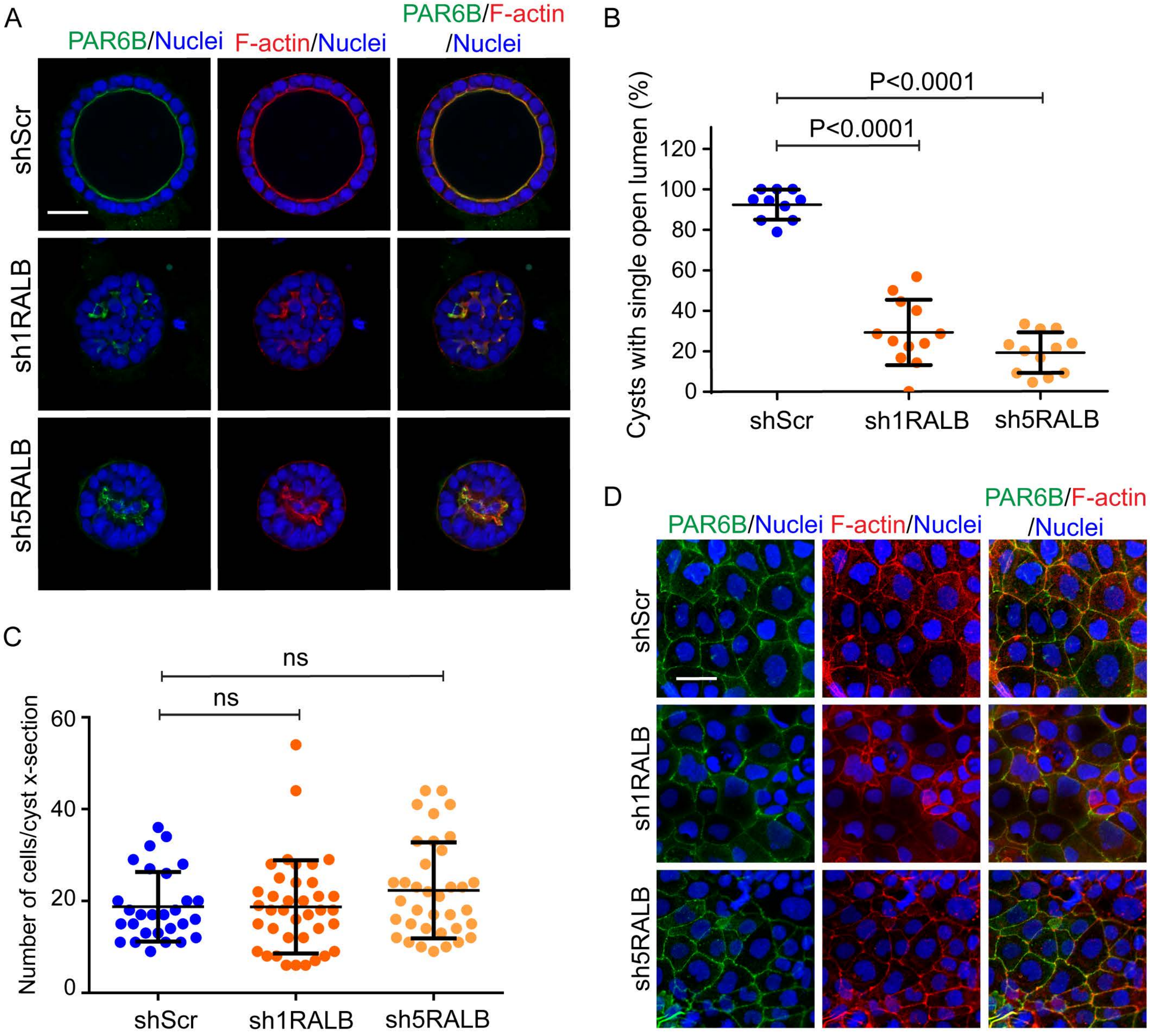

Supplementary Figure 4: RalB is required for lumen formation in Caco-2 spheroids. (A) Confocal images were captured for 3D shScr, sh1RALB, and sh5RALB knock-down Caco-2 spheroids cultured in semi-embedded BME and immunostained for PAR6B (green) and F-actin (red). (B) The quantification of the percentage of cysts with single open lumen in 3D shScr, sh1RALB, and sh5RALB knock-down Caco-2 spheroids. (C) The quantification of the number of cells per cross-section cyst in 3D shScr, sh1RALB, and sh5RALB knock-down Caco-2 spheroids. (D) Confocal images were captured for 2D shScr, sh1RALB, and sh5RALB knock-down Caco-2 cells immunostained for PAR6B (green) and F-actin (red). Scale Bars: A, 30μm; D, 50μm.

Wang-Supplementary Figure 5

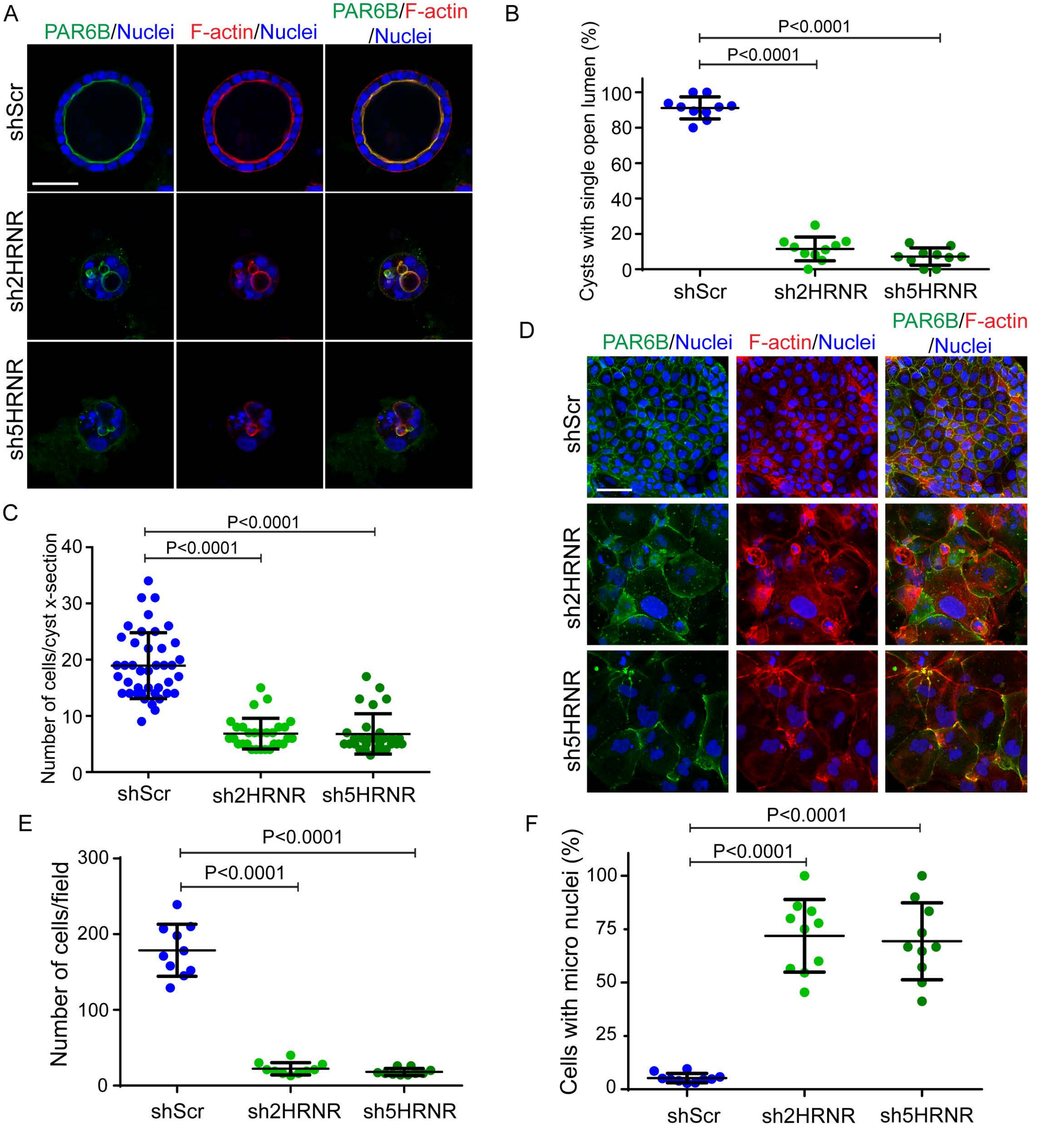

Supplementary Figure 5: HRNR is required for lumen formation in Caco-2 spheroids. (A) Confocal images were captured for 3D shScr, sh2HRNR, and sh5HRNR knock-down Caco-2 spheroids cultured in semi-embedded BME and immunostained for PAR6B (green) and F-actin (red). (B) The quantification of the percentage of cysts with single open lumen in 3D shScr, sh2HRNR, and sh5HRNR knock-down Caco-2 spheroids. (C) The quantification of the number of cells per cross-section cyst in 3D shScr, sh2HRNR, and sh5HRNR knock-down Caco-2 spheroids. (D) Confocal images were captured for 2D shScr, sh2HRNR, and sh5HRNR knock-down Caco-2 cells immunostained for PAR6B (green) and F-actin (red). (E) The quantification of the number of cells per field in 2D shScr, sh2HRNR, and sh5HRNR knock-down Caco-2 cells. (F) The quantification of the percentage of cells with micro nuclei in 2D shScr, sh2HRNR, and sh5HRNR knock-down Caco-2 cells. Scale Bars: A, 50μm; D, 100μm.

Wang-Supplementary Figure 6

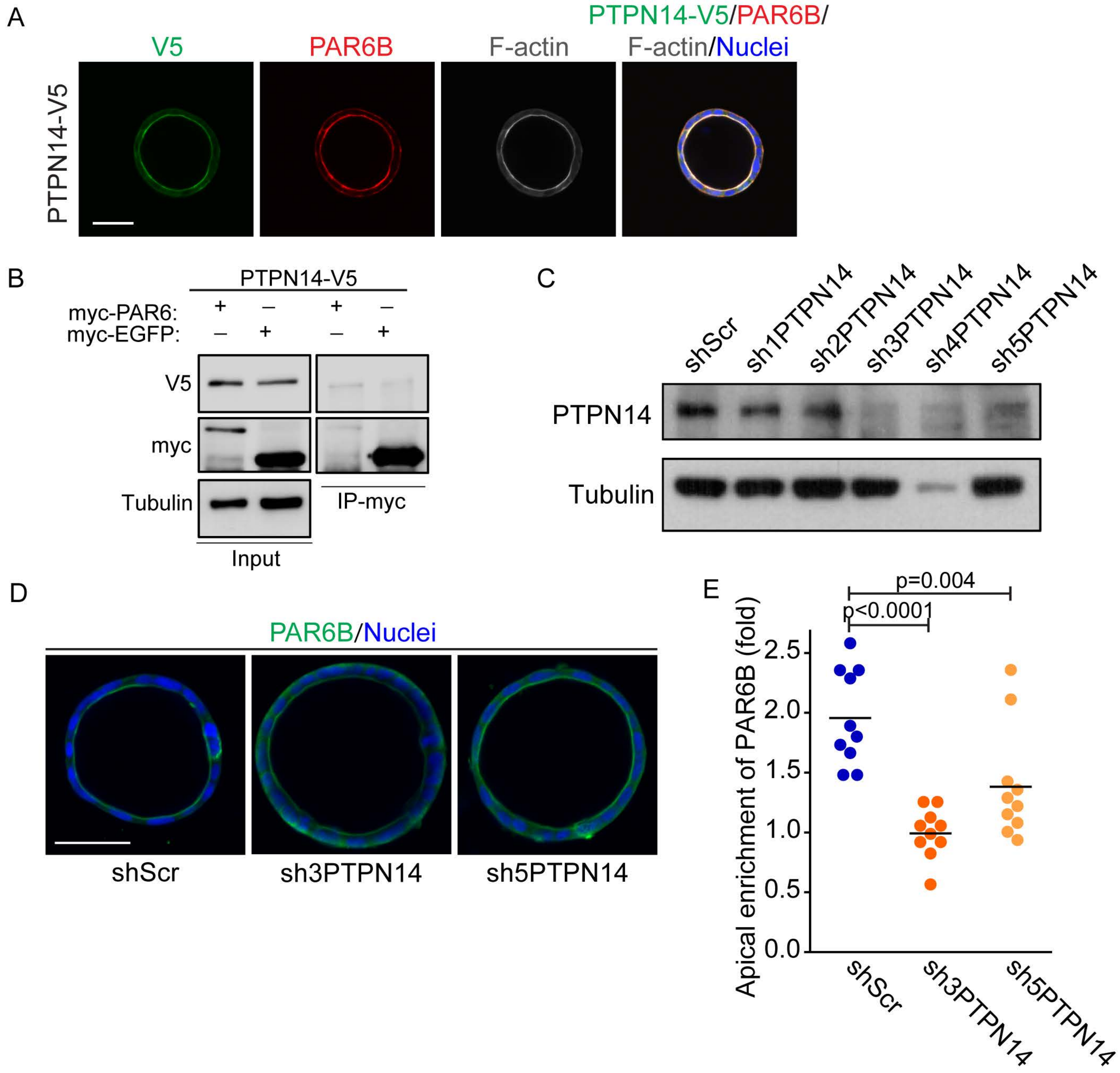

Supplementary Figure 6: PTPN14 is associated with PARD6B and localizes to apical membrane of polarized Caco-2 cysts. Associated with Figure 5. (A) Confocal images were captured for PTPN14-V5-expressed 3D Caco-2 cysts immunostained for PTPN14-V5 (green), Par6 (red), and F-actin (grey) show the co-localization of PTPN14 and PARD6B. (B) Co-immunoprecipitation of PTPN14-V5 and myc-EGFP or myc-PAR6B was performed with anti-myc in transient transfected HEK293 cells. The presence of PTPN14 in immunoprecipitates was determined by western blot analysis using anti-V5. (C) Western blot showing the knockdown efficiency of different shRNAs for PTPN14 in Caco-2 cells. (D) Confocal images show PAR6B (green) localization in shScr, sh3- and sh5-PTPN14 knock-down Caco-2 cysts. (E) Quantification of the fold change of apical enrichment of PAR6B in shScr, sh3- and sh5-PTPN14 knock-down Caco-2 cysts. Scale Bars: A, D, 50µm.

Wang-Supplementary Figure 7

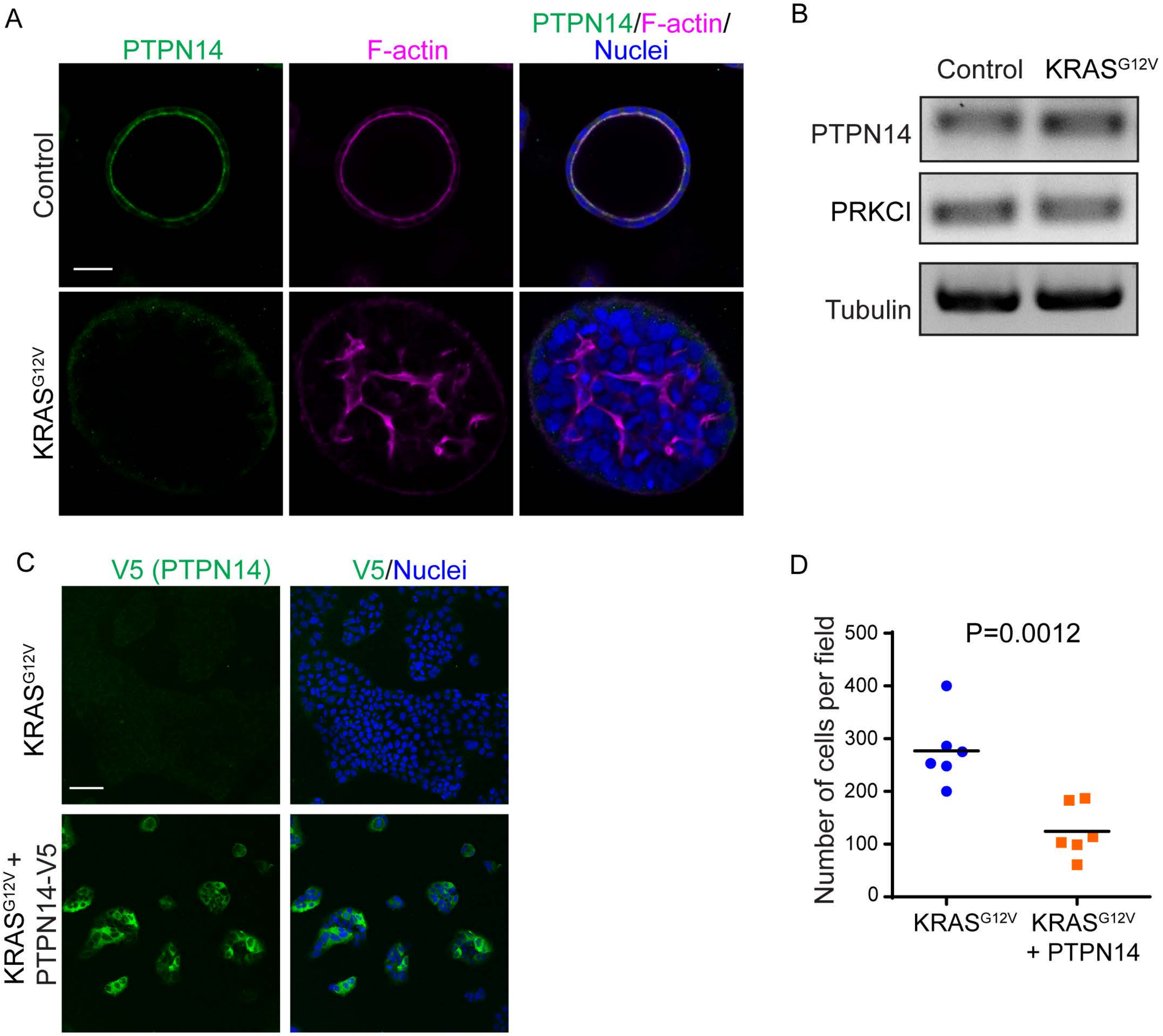

Supplementary Figure 7: PTPN14 suppresses KRAS^{G12V}-induced transformation in Caco-2 spheroids. Associated with Figure 6. (A) Confocal images were captured for control and KRAS^{G12V}-transformed Caco-2 spheroids immunostained for PTPN14 (green) and F-actin (magenta) show the expression and localization of PTPN14. (B) mRNA abundance of PTPN14 and PRKCI was compared between control and KRAS^{G12V}-transformed Caco-2 cells. (C) Images for PTPN14-V5 (green) showing overexpression of PTPN14 suppresses KRAS^{G12V}-induced transformation in 2D Caco-2 cells. (D) Quantification of number of cells per field in KRAS^{G12V}-transformed Caco-2 cells with or without PTPN14 overexpression. Scale Bars: A, 30μm; C, 100μm.

Wang-Supplementary Figure 8

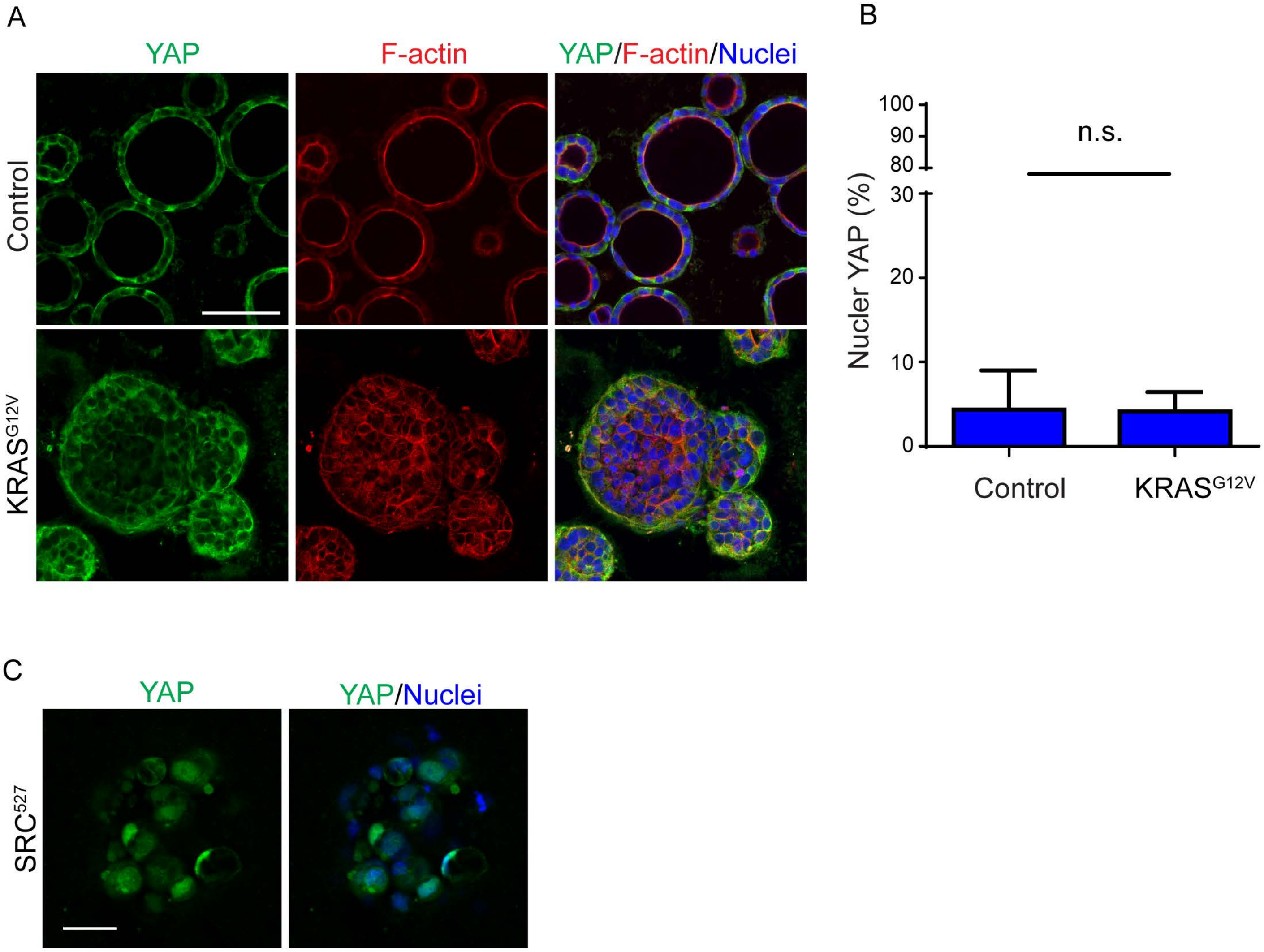

Supplementary Figure 8: PTPN14 is involved in other mechanisms instead of Hippo signalling in KRAS^{G12V}-induced transformation in Caco-2 cysts. Associated with Figure 6. (A) Confocal images for YAP (green) and F-actin (red) shows the localization of YAP in control and KRAS^{G12V}-transformed Caco-2 spheroids. (B) Quantification the percentage of nuclear YAP in control and KRAS^{G12V}-transformed Caco-2 spheroids. (C) Confocal images for YAP (green) showing YAP translocates to the nucleus in SRC^{527}-expressed Caco-2 spheroids. Scale Bars: A, 100μm; C, 50μm.

Fig. 2B

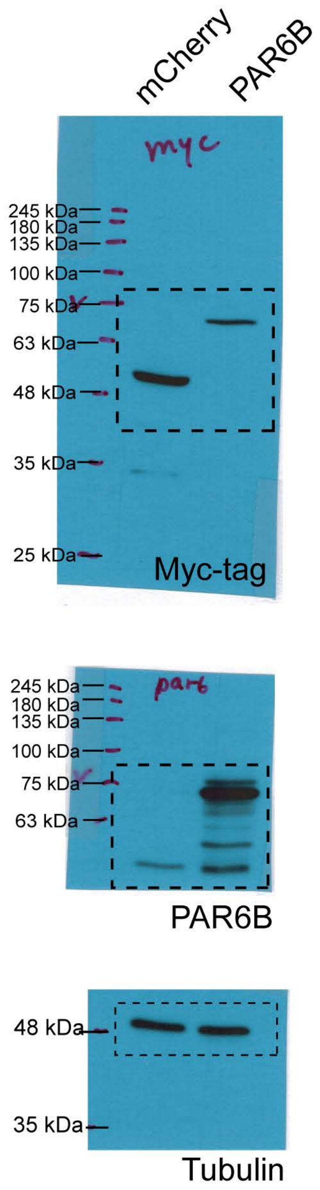
mCherry
PAR6B
myc
245 kDa
180 kDa
135 kDa
100 kDa
75 kDa
63 kDa
48 kDa
35 kDa
25 kDa
Myc-tag
par6
245 kDa
180 kDa
135 kDa
100 kDa
75 kDa
63 kDa
PAR6B
48 kDa
35 kDa
Tubulin

Fig. 2C

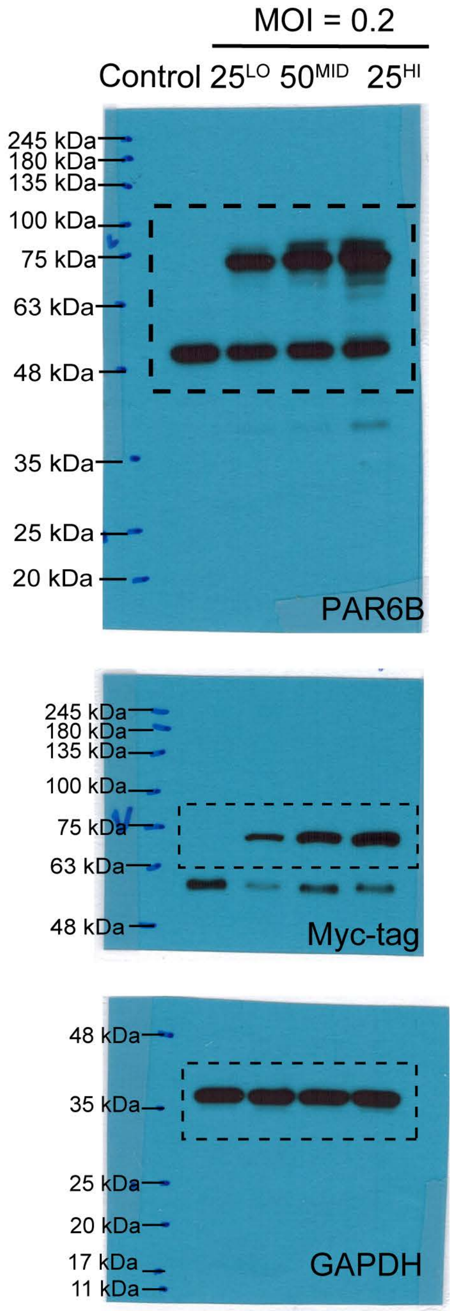
MOI = 0.2
Control 25LO 50MID 25HI
245 kDa
180 kDa
135 kDa
100 kDa
75 kDa
63 kDa
48 kDa
35 kDa
25 kDa
20 kDa
PAR6B
245 kDa
180 kDa
135 kDa
100 kDa
75 kDa
63 kDa
48 kDa
Myc-tag
48 kDa
35 kDa
25 kDa
20 kDa
17 kDa
11 kDa
GAPDH

Fig. 4A

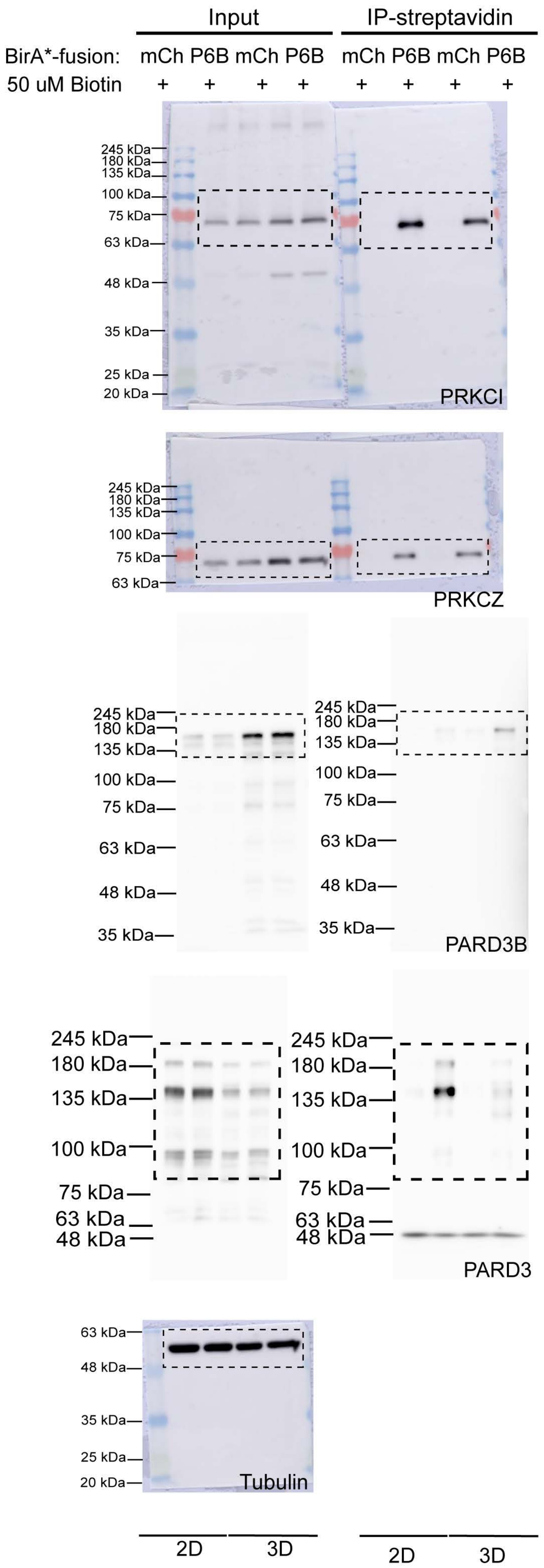
Input
IP-streptavidin
BirA*-fusion: mCh P6B mCh P6B mCh P6B mCh P6B
50 uM Biotin + + + + + + + +
245 kDa
180 kDa
135 kDa
100 kDa
75 kDa
63 kDa
48 kDa
35 kDa
25 kDa
20 kDa
PRKCI
PRKCZ
PARD3B
PARD3
Tubulin
2D
3D
2D
3D

Fig. 4B

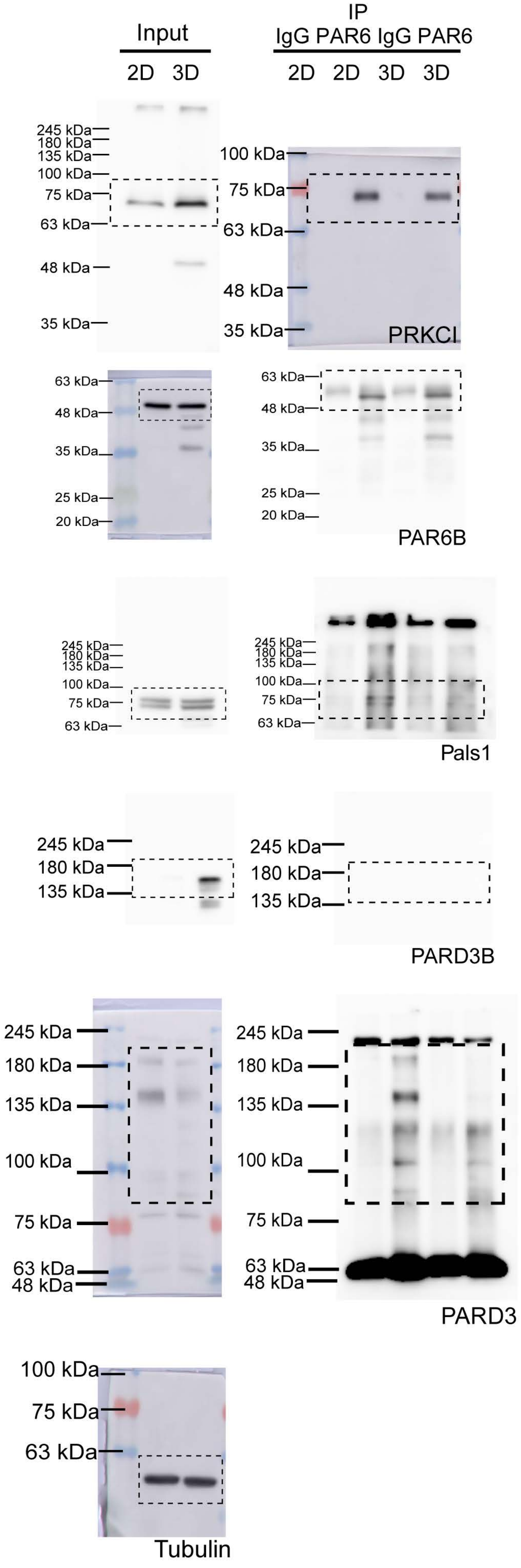
Input
2D 3D
IP
IgG PAR6 IgG PAR6
2D 2D 3D 3D
245 kDa
180 kDa
135 kDa
100 kDa
75 kDa
63 kDa
48 kDa
35 kDa
25 kDa
20 kDa
PRKCI
PAR6B
Pals1
PARD3B
PARD3
Tubulin

Fig. 5C

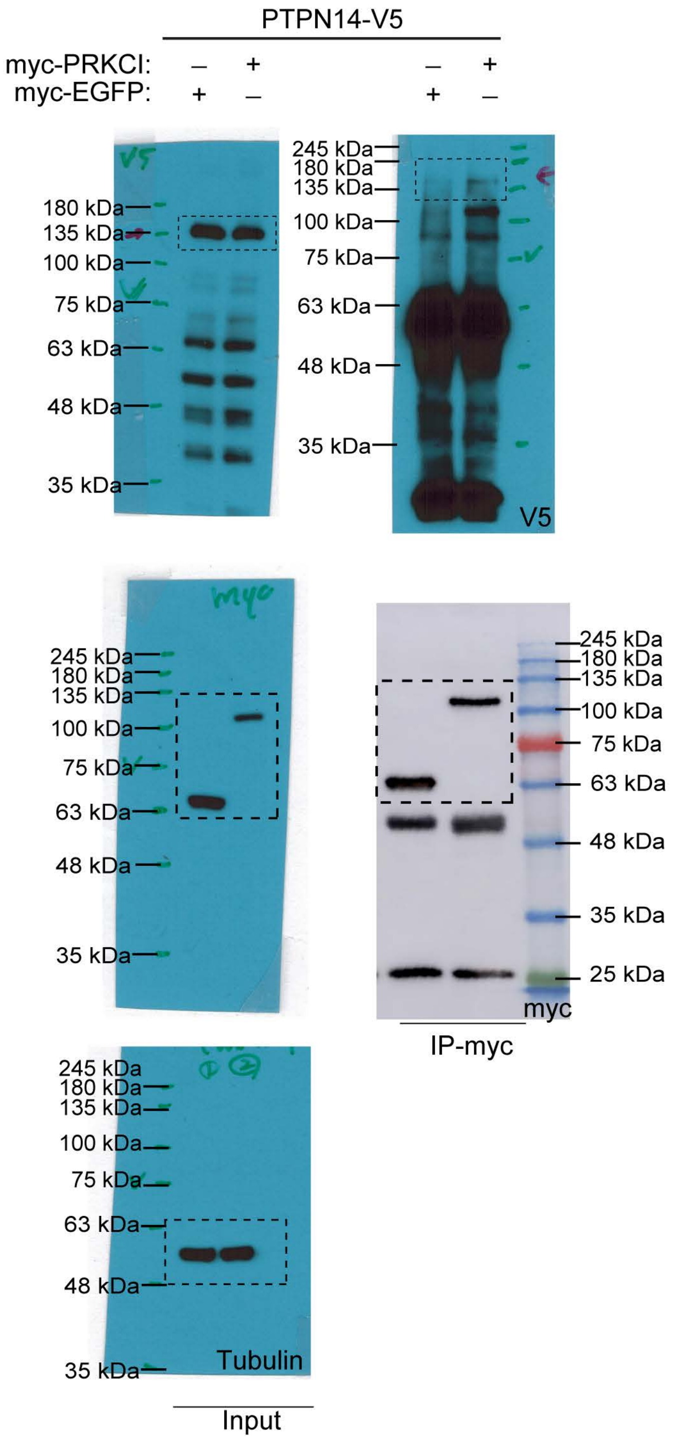
PTPN14-V5
myc-PRKCI: – + – +
myc-EGFP: + – + –
180 kDa
135 kDa
100 kDa
75 kDa
63 kDa
48 kDa
35 kDa
245 kDa
180 kDa
135 kDa
100 kDa
75 kDa
63 kDa
48 kDa
35 kDa
V5
245 kDa
180 kDa
135 kDa
100 kDa
75 kDa
63 kDa
48 kDa
35 kDa
245 kDa
180 kDa
135 kDa
100 kDa
75 kDa
63 kDa
48 kDa
35 kDa
25 kDa
myc
IP-myc
245 kDa
180 kDa
135 kDa
100 kDa
75 kDa
63 kDa
48 kDa
35 kDa
Tubulin
Input

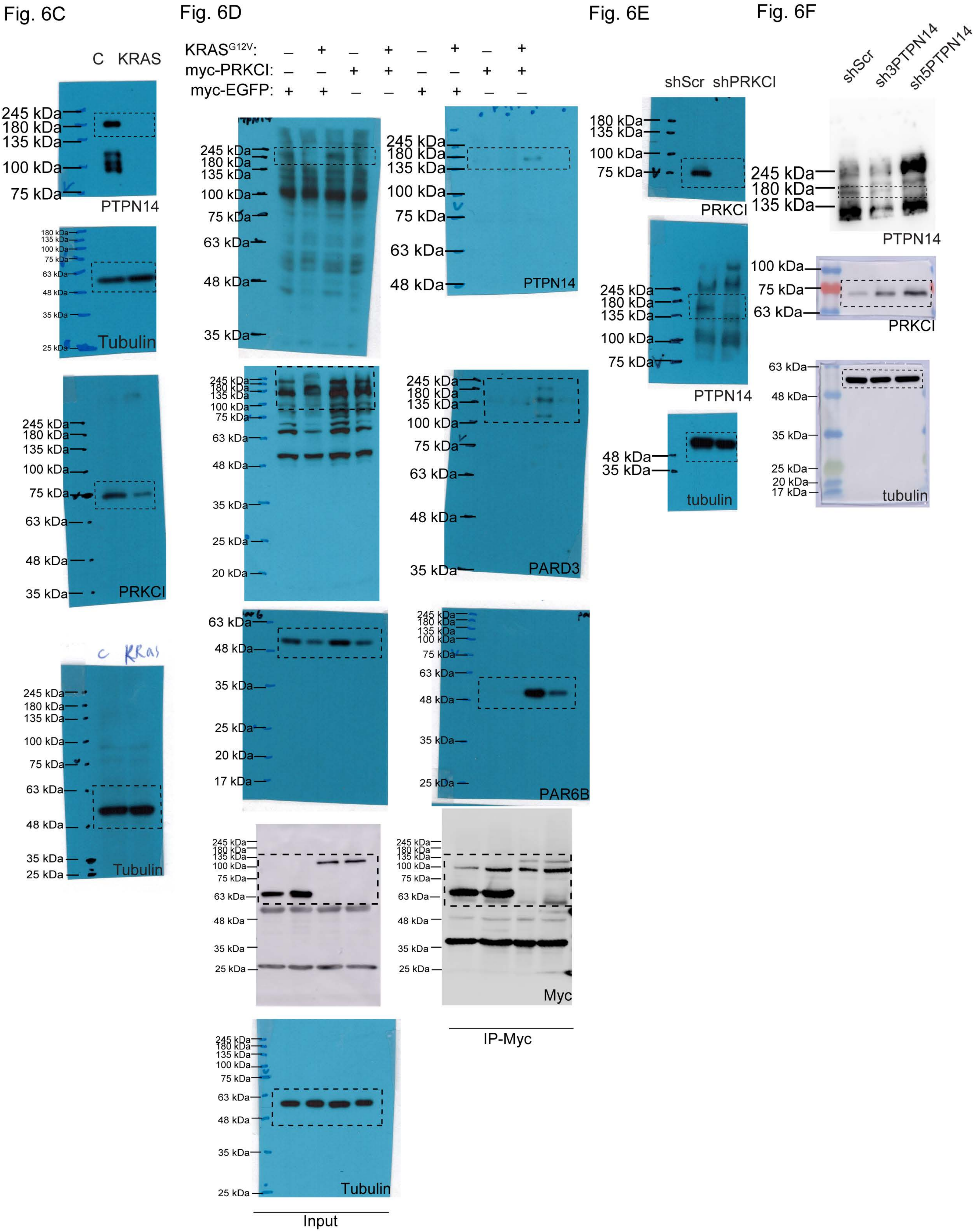
Fig. 6C
C KRAS
PTPN14
Tubulin
PRKCI
c KRas
Tubulin
Fig. 6D
KRAS^{G12V}: – + – + – + – +
myc-PRKCI: – – + + – – + +
myc-EGFP: + + – – + + – –
PTPN14
PARD3
PAR6B
Myc
Tubulin
Input
IP-Myc
Fig. 6E
shScr shPRKCI
PRKCI
PTPN14
tubulin
Fig. 6F
shScr
sh3PTPN14
sh5PTPN14
PTPN14
PRKCI
tubulin
245 kDa
180 kDa
135 kDa
100 kDa
75 kDa
63 kDa
48 kDa
35 kDa
25 kDa
20 kDa
17 kDa

Supplementary Fig. S3A

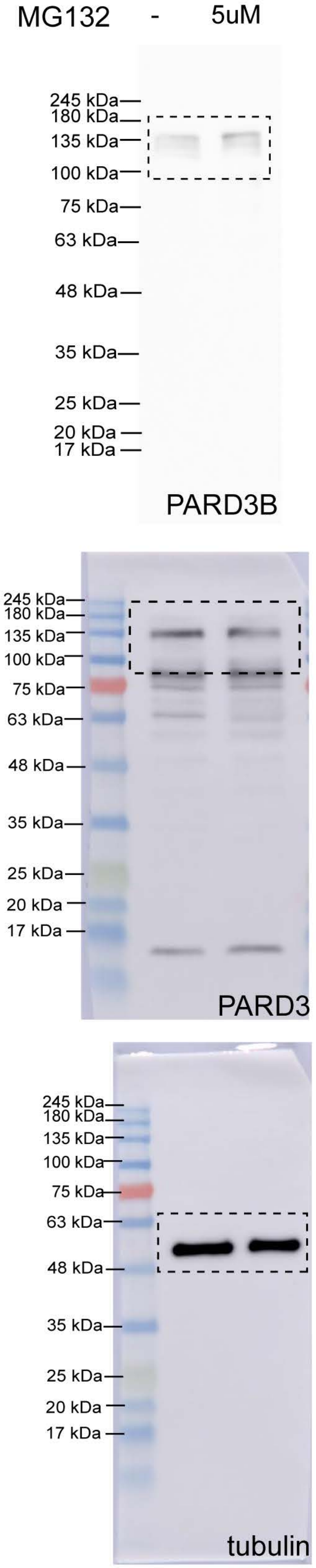

Supplementary Fig. S3B

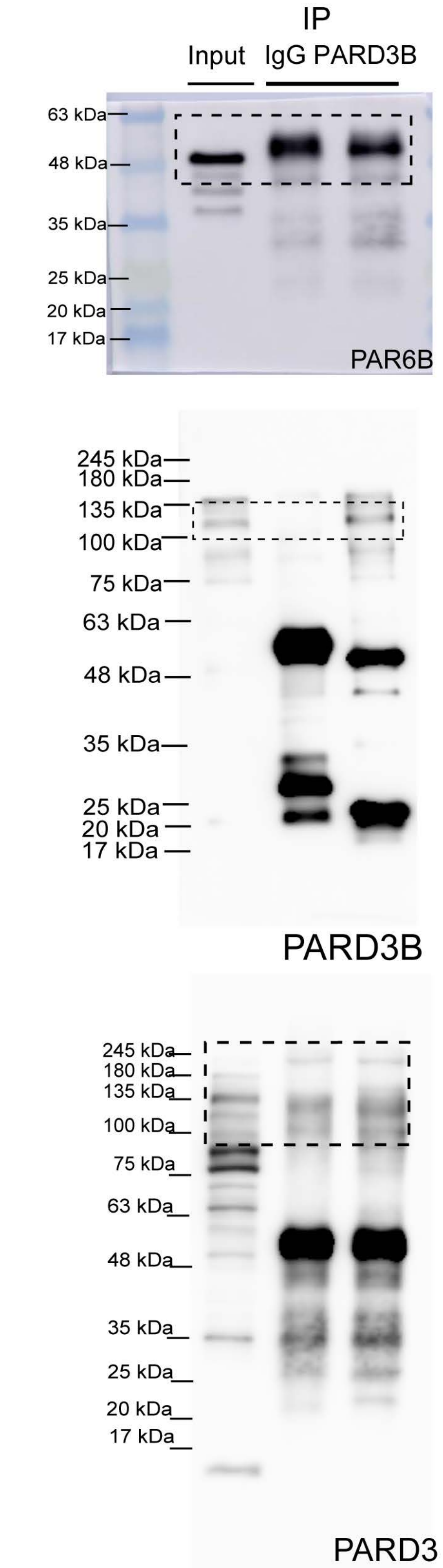

Supplementary Fig. S6B

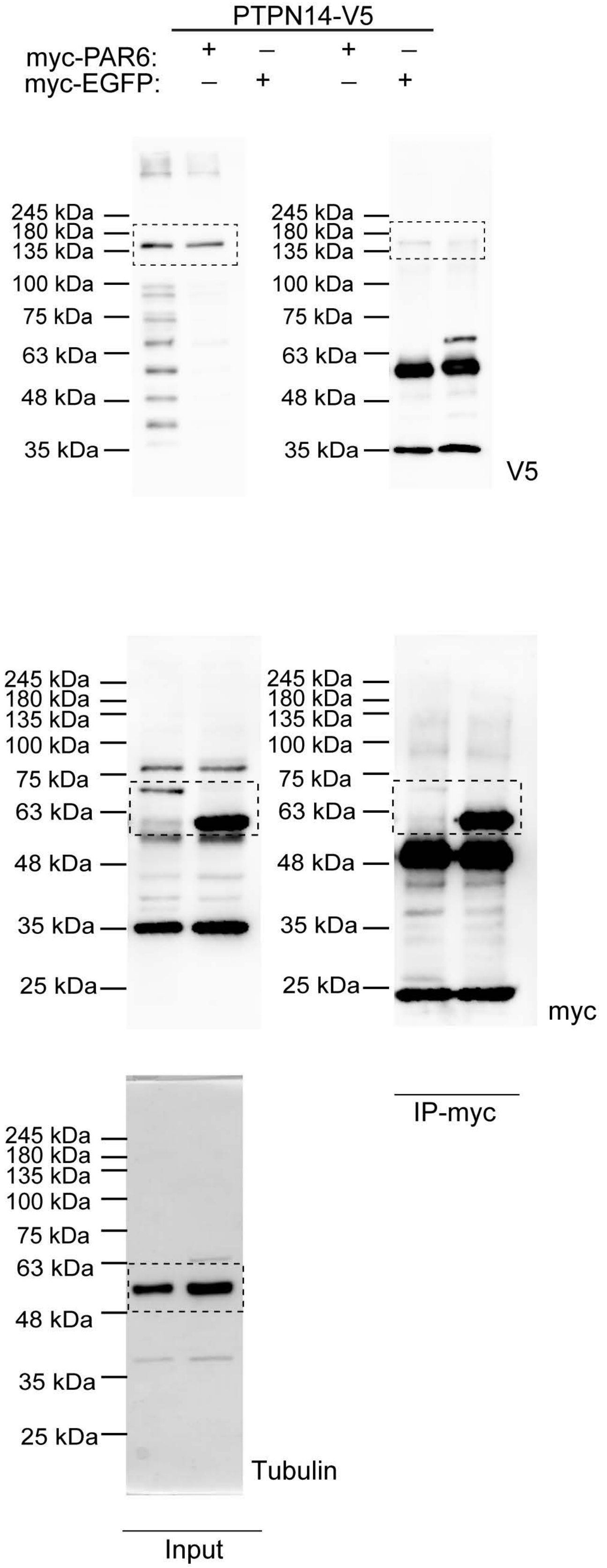

Supplementary Fig. S6C

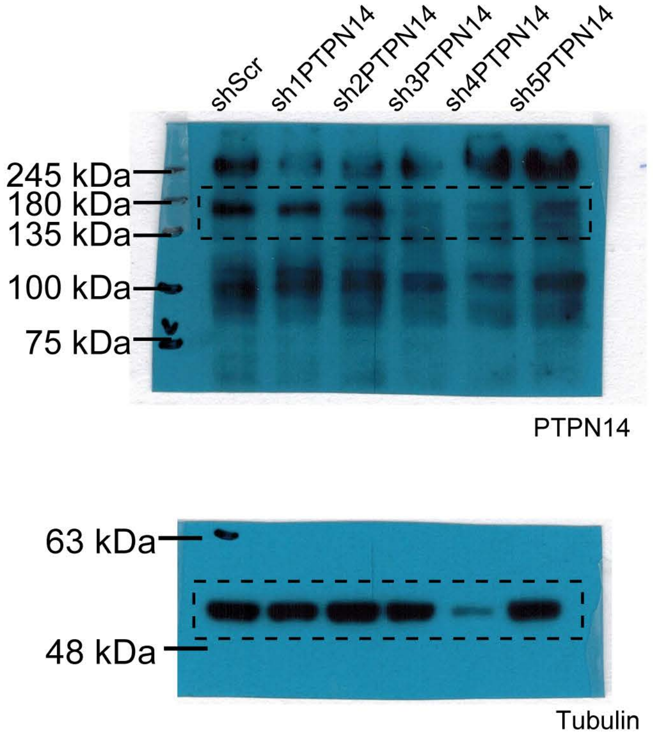

Supplementary Fig. S7B

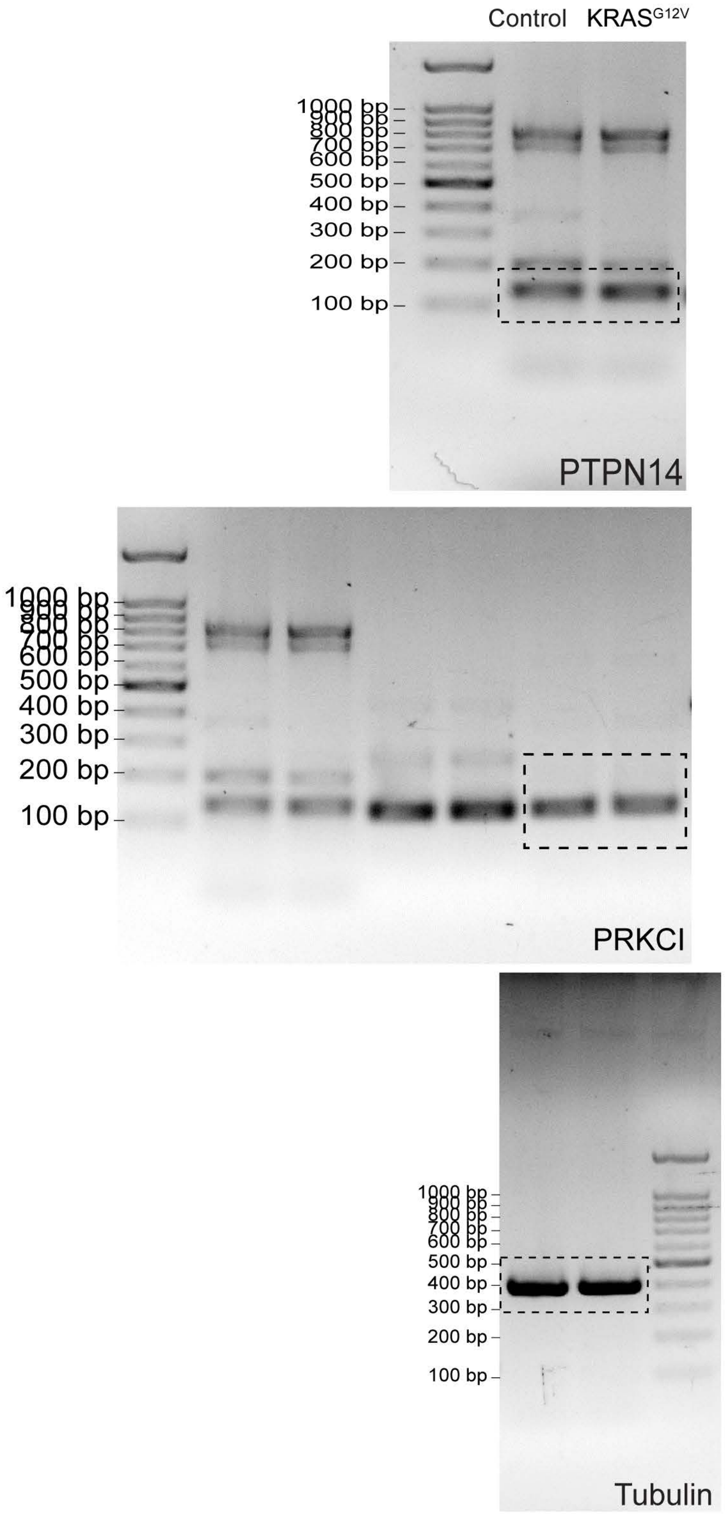

Supplement: Supplementary file 1 — Supplementary Information. [file 41598_2021_2178_MOESM1_ESM.pdf]
